# In utero air pollution exposure and pre- and postnatal brain development: a review

**DOI:** 10.1016/j.nsa.2026.107019

**Published:** 2026-07-11

**Authors:** Vera Goossens, Nora L. Großmann, Ulrike Gehring, Mireille N. Bekker, Hilleke E. Hulshoff Pol, Sonja M.C. de Zwarte

**Affiliations:** a Department of Experimental Psychology, Helmholtz Institute, Utrecht University, Utrecht, the Netherlands; b Department of Developmental Psychology, Utrecht University, Utrecht, the Netherlands; c Institute for Risk Assessment Sciences, Utrecht University, Utrecht, the Netherlands; d Department of Obstetrics, University Medical Center Utrecht, Utrecht, the Netherlands; e Department of Psychiatry, UMC Brain Center, University Medical Center Utrecht, Utrecht University, Utrecht, the Netherlands

**Keywords:** Air pollution, Prenatal exposure, Brain development, Fetal ultrasound, MRI

## Abstract

Air pollution is a major threat to public health globally and may also adversely influence brain development. Key neurodevelopmental processes unfold within narrow and sensitive windows during fetal life and continue through childhood and adolescence, leaving the brain particularly vulnerable to environmental influences. Prenatal air pollution exposure could possibly disrupt fetal neurodevelopmental processes via direct placental crossing of pollutants or through maternal inflammatory processes. This literature review evaluates evidence from ultrasound and MRI studies examining associations of *in utero* air pollution exposure (particulate matter (PM_10_ (≤10 μm), PM_coarse_ (10 – 2.5 μm), PM_2.5_ (≤2.5 μm), PM characteristics (PM composition and oxidative potential)), nitrogen oxides (NO_x_), sulfur dioxide (SO_2_), carbon monoxide (CO), black carbon, and ozone (O_3_)) with structural brain development from the fetal period through adolescence. Findings are summarized per pollutant to evaluate potential adverse effects of individual pollutants in the complex mixture of air pollution. Results indicate associations between *in utero* air pollution exposure and structural brain outcomes, although no distinct patterns or differences between individual pollutants could be identified. Prenatal ultrasound studies reported negative associations between *in utero* air pollution exposure levels and the global fetal brain metrics biparietal diameter and head circumference in geographical regions with relatively high air pollution levels. In contrast, childhood and adolescent MRI studies, mainly conducted in relatively low exposure areas, showed no associations with global brain volumes. However, associations with regional brain measures were reported including changes in cerebral cortex regions, subcortical gray matter structures, and global and regional white matter structures, potentially impacting cognitive and behavioral outcomes in childhood. Overall, current evidence supports a link between prenatal air pollution and altered brain development, highlighting the need for longitudinal and mechanistic studies to clarify causal pathways and functional consequences.

## 1 Introduction

The involuntary exposure to air pollution affects nearly the entire global population across all life stages and causes multiple adverse health effects, including pulmonary and cardiovascular morbidity and mortality, type II diabetes, adverse reproductive outcomes, and central nervous system effects (Block et al., 2012; Boogaard et al., 2019; Forman and Finch, 2018; Health Effects Institute, 2025). Growing evidence further suggests that prenatal exposure to air pollution can adversely affect the developing brain, with neurotoxic effects being described in human brain, animal brain and *in vitro* studies (Costa et al., 2014, 2019; Lopuszanska and Samardakiewicz, 2020). Air pollution comprises a complex mixture of solid particles, liquid droplets, and gasses, varying by region and source (Block et al., 2012; Costa et al., 2020; Künzli et al., 2010). Particles vary in size and composition. As biological activity differs between pollutants (Jankowska-Kieltyka et al., 2021; Rodier, 1995), *in utero* exposure may therefore exert heterogeneous effects on the fetal brain, depending on the specific pollutant profile.

**Fig. 1:**
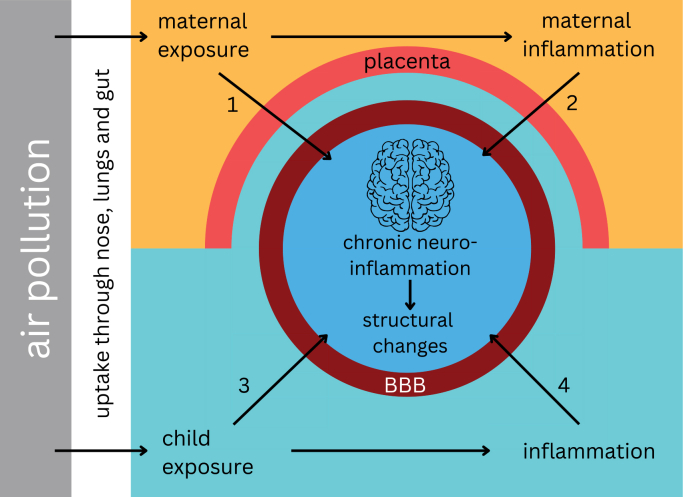
**Schematic and simplified overview of the hypothesized biological mechanistic pathways of air pollution affecting brain development.** Maternal exposure (top; yellow) impacts the brain prenatally when pollutants themselves (direct pathway; 1) or inflammation markers (indirect pathway; 2) cross the placenta and subsequently the blood-brain barrier (BBB). After birth (bottom; blue) air pollutants are directly taken up by the child and pass the BBB (direct pathway; 3) or cause systemic inflammation (indirect pathway; 4). All pathways are hypothesized to contribute to chronic neuroinflammation, potentially causing structural changes in the brain.

During the prenatal period, the brain is particularly vulnerable to external adversities. Critical neurodevelopmental processes occur within this relatively short time window, while natural barriers, such as the blood-brain barrier, are not yet fully developed (Rice and Barone, 2000; Sunyer and Dadvand, 2019). Consequently, relatively small disruptions in these critical processes can have a large impact on the developing brain (Forman and Finch, 2018; Behura et al., 2019; Fu et al., 2019; Knuesel et al., 2014; Yi et al., 2022). Several biological mechanisms that may underlie the impact of air pollutants on early brain development have been explored. Maternal exposure to air pollution can affect fetal brain development either indirectly or directly (Fig. 1). Maternal uptake of pollutants primarily occurs through the lungs, but also through the olfactory system, gut and/or possibly even via the eyes (Forman and Finch, 2018; Costa et al., 2019; Rodier, 1995; Armas and D'Angiulli, 2022; Herting et al., 2019) and, depending on size, the smallest particle fraction (so-called ultrafine particles <100 nm, UFP) can subsequently translocate to the maternal circulation.

In the indirect pathway, maternal air pollution exposure induces systemic inflammation, which may result in placental impairment as well as inflammatory cytokines crossing the placenta and subsequently influencing fetal brain development (Behura et al., 2019; Ha et al., 2019; Jonakait, 2007; Mortamais et al., 2019;Jiang et al., 2018). The direct pathway posits that relatively small air pollutants, specifically UFPs, themselves can reach the placenta and the fetus. Once they have entered the maternal circulation, these pollutants may escape phagocytosis by the liver and circulating macrophages, and ultimately, cross the placenta (Forman and Finch, 2018; Herting et al., 2019). Recent studies reported evidence of black carbon particles being able to cross the placenta and subsequently translocate to fetal organs (Bongaerts et al., 2022; Bové et al., 2019). Pollutants can potentially disrupt a range of processes in the placenta itself and, when crossing the placenta, can also act directly on fetal tissues and organs, possibly resulting in neurodevelopmental consequences (Costa et al., 2020; Yi et al., 2022; Fowler et al., 2023; Künzli et al., 2006). However, the extent to which air pollution influences fetal human brain development, the pollutants that are primarily responsible , and the subsequent potential long-term effects, remain largely unresolved.

Investigating the effects of *in utero* air pollution exposure on brain structure and early development relies primarily on anthropometric measurements (common in the neonatal and infant periods) and neuroimaging studies. Evidence for pollutant-specific effects on early brain development comes from a meta-analysis which reported that higher prenatal exposure to particulate matter (PM) ≤ 2.5 μm (PM_2.5_) was associated with a smaller head circumference (HC) at birth, whereas nitrogen dioxide (NO_2_) was associated with smaller birth length only (i.e., a non-specific brain-related outcome) (Fu et al., 2019). Notably, the same meta-analysis found no significant association between *in utero* air pollution exposure and fetal HC as measured by ultrasound imaging, while results for another common fetal biometric measure, biparietal diameter (BPD), remained inconclusive.

Several studies have assessed the long-term consequences of prenatal air pollution exposure on neurodevelopment using magnetic resonance imaging (MRI), with findings recently summarized in three systematic reviews (Fowler et al., 2023; Morrel et al., 2024.; Parenteau et al., 2024). These reviews reported no significant associations between *in utero* air pollution exposure and global brain structure in childhood or adolescence, while observations for specific regions of interest (among which e.g., corpus callosum, hippocampus, prefrontal cortex) showed mixed results depending on exposure time, pollutant, and age at MRI (Fowler et al., 2023; Morrel et al., 2024.; Parenteau et al., 2024). All three reviews encompassed multiple exposure windows spanning the *in utero*, childhood and adolescent periods along with varying pollutant-specific approaches and did not specifically focus on effects of prenatal exposure. Given this broad scope and the diversity of imaging modalities employed, no comprehensive studies to date have synthesized brain structural changes from the fetal period through adolescence specifically in relation to *in utero* exposure to specific air pollutants.

The aim of this literature review was therefore to provide a summary of the evidence for potential influences of *in utero* air pollution exposure on the developing central nervous system from studies published up to February 25, 2026. The review provides a complete overview of imaging studies investigating the effects of *in utero* air pollution exposure on brain development from the fetal period up to adolescence and discusses it in light of new evidence. The focus is exclusively on *in utero* air pollution exposure, aiming to disentangle potential effects on the fetus from longer-term childhood and adolescent consequences of *in utero* exposure on the developing brain. Findings are discussed per pollutant to investigate possible differences in biological activity.

## 2 Methods

### 2.1 Data sources

Brain imaging studies investigating the association between *in utero* air pollution exposure and structural brain changes were obtained through an extensive PubMed and Google Scholar search (initial search by VG, verification by NG). The search terms were (“*in utero*” OR “prenatal” OR “fetal” OR ‘‘maternal’’ OR ‘‘pregnancy’‘) AND (“air pollution”) AND (“brain” OR ‘‘neurodevelopment’‘) AND (“imaging” OR “ultrasound” OR “MRI” OR ‘‘head circumference’’ OR ‘‘biparietal diameter’‘). Titles and abstracts were examined to see whether they fulfilled the inclusion criteria as described in the next paragraph. The search was complemented by examining the reference list of the primary and review articles found on air pollution exposure and structural brain outcomes.

### 2.2 Study selection

Studies were included if they were available in English, published in peer-reviewed journals, and if they investigated the effect of *in utero* air pollution exposure on human brain structure measured either i) during pregnancy or ii) after birth in children until the age of 18. Ultrasound, and structural and diffusion MRI modalities (no criteria were set for regions of interest) were included to quantify brain structure. Prenatal neuroimaging studies published between January 1, 2017, and February 25, 2026, were included to give an updated overview of the literature, building on the previously published systematic review and meta-analysis on publications up to July 2017 (Fu et al., 2019). In addition, neuroimaging studies in children and adolescents aged 0-17 years and published between January 1, 2000, and February 25, 2026, were included. Previous systematic reviews have shown that no neuroimaging studies investigating the relationship between air pollution and brain structure in children were published before 2000 (Morrel et al., 2024.; Parenteau et al., 2024). Only studies that investigated outdoor air pollution exposure during the prenatal period (any time between conception until birth) and reported associations for a specified increase in individual pollutant concentrations were included. No criteria were set for sample size, type, level or number of pollutants measured. Brain imaging studies primarily focusing on interaction effects between air pollution and other risk factors were excluded.

### 2.3 Data extraction

First author, year of publication, sample size, (gestational) age at brain measurement, gestational age at exposure measurement, type of air pollutant(s), average exposure levels per pollutant and type of outcome measurement were extracted from each article that met the inclusion criteria (initially by VG and verified by NG). Discrepancies between reviewers were resolved by discussion. Results of both single-pollutant and multi-pollutant models were extracted. Results adjusted for at least (gestational) age and sex were prioritized over crude results if both were reported. The quality of the studies and risk of bias were assessed using the Newcastle–Ottawa scale.

## 3 Results

**Fig. 2:**
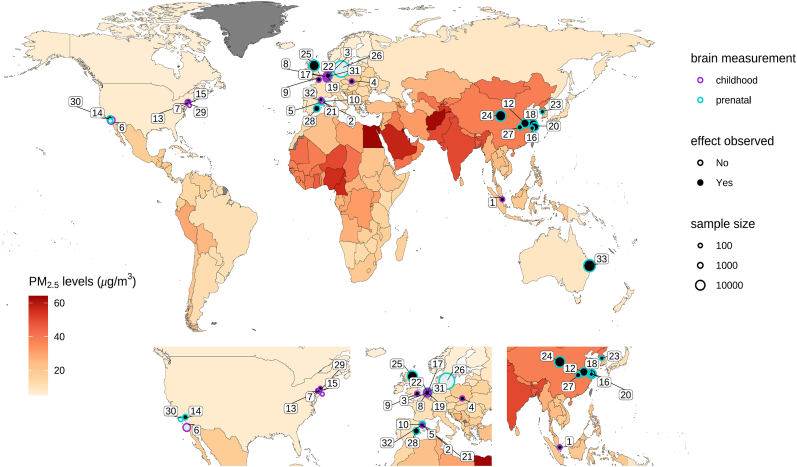
**Neuroimaging studies on *in utero* exposure to air pollution and early brain development across the world.** Color coding on the world map is based on annual mean particulate matter (≤2.5 μm) (PM_2.5_) concentration per country only as an example to visualize global air pollution differences. All imaging studies discussed in this review and in Fu et al. ‘s meta-analysis (Fu et al., 2019) are included, visualizing sample size (dot size) and whether significant associations were observed with any air pollutant prenatally (blue) or in childhood (pink): 1. Buthmann et al. (2025); 2. Gómez-Herrera et al., (2025); 3. Kusters et al., (2025); 4. Lewandowska et al. (2025); 5. Pujol et al. (2025) 6. Szwed et al., (2025); 7. Yang et al. (2025); 8. Kusters et al. (2024); 9. Bos et al. (2023); 10. Chen et al. (2023); 11. Leung et al. (2023); 12. Li et al. (2022); 13. Margolis et al. (2022); 14. Peterson et al. (2022); 15. Peterson et al. (2022); 16. Cao et al. (2021); 17. Lubczyńska et al. (2021); 18. Zhao et al. (2021); 19. Lubczyńska et al. (2020); 20. Cao et al. (2019); 21. Mortamais et al. (2019); 22. Guxens et al. (2018); 23. Lamichhane et al. (2018); 24. Zhao et al. (2018); 25. Clemens et al. (2017); 26. Malmqvist et al. (2017); 27. Wang et al., (2017); 28. Iñiguez et al. (2016); 29. Peterson et al. (2015); 30. Ritz et al. (2014); 31. van den Hooven et al., 2012; 32. Aguilera et al. (2010); 33. Hansen et al. ( 2008). Color coding is based on annual mean PM_2.5_ concentration per country and does not represent within-country variation. The annual mean PM_2.5_ concentration in urban areas (measured from fixed-site, population-oriented monitors located within the metropolitan areas; estimated using data integration from satellite remote sensing, population estimates, topography and ground measurements) is obtained from https://www.who.int/data/gho/data/themes/air-pollution/who-air-quality-database.

**Fig. 3:**
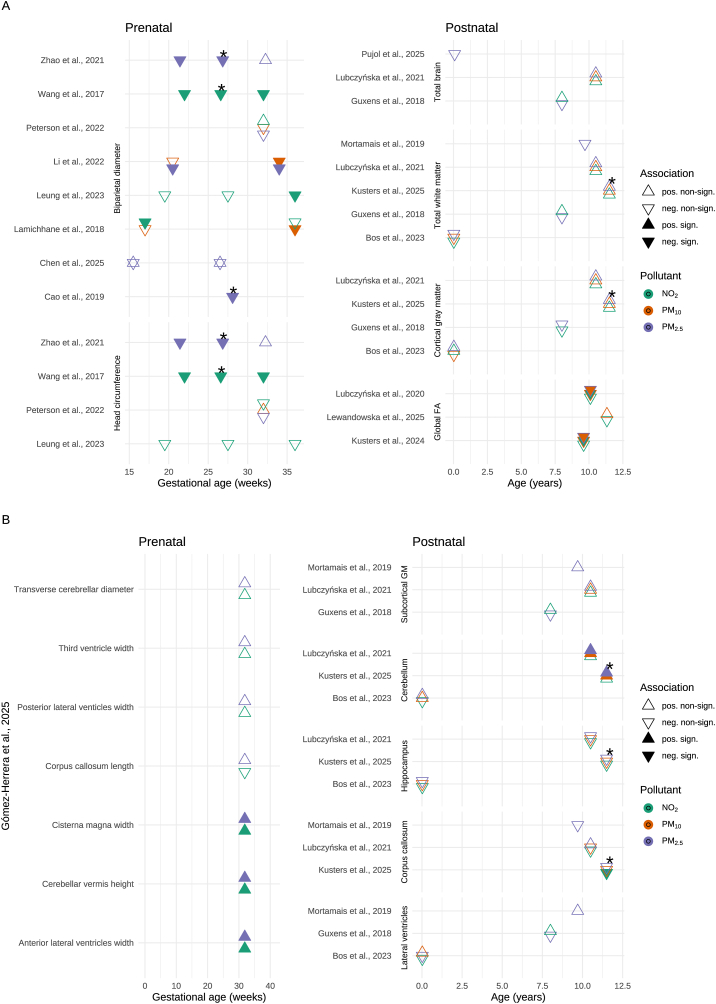
***In utero* air pollution and A) global brain measures and B) subcortical brain measures.** Shown is the direction of effect per study for global brain structure *in utero* (left) and during childhood and adolescence (right) associated with *in utero* air pollution. The triangles indicate the direction of the effect, and solid shapes indicate that the effect is significant. The shapes are color coded: NO_2_ (green), PM_2.5_ (purple), and PM_10_ (red) *in utero* air pollution exposure. Only brain measures that are included in at least three of the reviewed studies are depicted (except for regional prenatal measures shown in B on the left side). Studies that reported only on growth velocity were not included. Age represents mean or median age of the study population if provided; if not provided, the median of the provided age-range was calculated. ∗ indicates studies using repeated measures. FA: fractional anisotropy; GM: gray matter.

Our search identified 26 articles that matched the inclusion criteria. Prenatal imaging studies (N = 11) investigating the effect of *in utero* air pollution exposure solely used ultrasound as modality, while all postnatal brain imaging studies (N = 15) were exclusively MRI studies. Therefore, prenatal and postnatal imaging studies are reviewed and summarized separately. The studies are all prospective cohort studies, except for one study (Leung et al., 2023), which is a retrospective cohort study. In the results section and in Fig. 2, we review the significant findings (*p* < 0.05) of all included studies. Further findings (including non-significant results for HC and BPD, and for most frequently investigated exposure-MRI measure pairs) are summarized in Fig. 3 and Tables S1–S4. Due to the exploratory nature of many MRI studies that often investigate many regions of interest, we limited the summary of non-significant results to most commonly investigated air pollutants (Table S2).

For both prenatal and postnatal imaging studies, the results are reviewed per pollutant. The investigated pollutants included PM, sulfur dioxide (SO_2_), carbon monoxide (CO), ozone (O_3_), black carbon, NO_2_, and nitrogen oxides (NO_x_). PM is classified based on particle diameter: PM_10_ (≤10 μm), PM_coarse_ (2.5 - 10 μm), PM_2.5_ or fine particles (≤2.5 μm), and PM_0.1_ or ultrafine particles (UFP, ≤0.1 μm). It is important to note that PM_10_ contains all particles smaller than 10 μm and thus includes PM_2.5_, PM_coarse_ and UFPs (Rodier, 1995; Brockmeyer and D'Angiulli, 2016). PM is a complex mixture of particles with regional and source specific differences in composition. The investigated components included polycyclic aromatic hydrocarbons (PAHs), benzo[a]pyrene (B[a]P), organic carbon (OC), PM_2.5_ absorbance (proxy for elemental carbon) and the inorganic components copper (Cu), iron (Fe), potassium (K), silicon (Si) and zinc (Zn). The oxidative potential (OP) of PM_2.5_ was also investigated, which is a quantification of the ability of PM_2.5_ to induce oxidative stress. OP was evaluated using either dithiothreitol (OP_DTT_) or electron spin resonance (OP_ESR_). Exposure is commonly expressed as mass concentration (μg/m^3^), except for UFP, for which exposure is expressed as particle number concentration due to its relatively low mass (Brockmeyer and D'Angiulli, 2016).

Next to investigating the effects of each pollutant independently, some studies explored multi-pollutant approaches. Apart from adjustment for other pollutants, four other multi-pollutant approaches were applied in the reviewed studies: the deletion/substitution/addition (DSA) algorithm, canonical correlation analysis (CCA), Least Absolute Shrinkage and Selection Operator (LASSO), and ridge regression. The DSA algorithm is an iterative linear regression model search algorithm to select the variables most predictive of the outcome. In each iteration, a term may be removed from, replaced in or added to the model (Agier et al., 2016). CCA is a multidimensional statistical method that looks for a weighted linear composite for two sets of variables that maximizes their correlation while addressing multicollinearity (Bos et al., 2023). LASSO is a shrinkage method that identifies variables that explain the most variance in the outcome. It performs variable selection by attributing zero values to the lowest regression coefficients and thus retains only the most informative predictors (Agier et al., 2016). Ridge regression shrinks the coefficients of correlated predictors equally without eliminating them and ensures numerical stability (Agier et al., 2016; Friedman et al., 2010).

Using the Newcastle–Ottawa quality assessment scales, we assessed risk of bias in selection, comparability, and outcome (Tables S5 and S6 for studies investigating fetal and postnatal brain development, respectively). Since all studies passed at least six out of the eight assessed points, we did not identify any risk of bias in the included cohorts. Most studies included participants that represented the studied community adequately. Participants with different levels of exposure were drawn from the same community in all studies, and all studies used adequate exposure assessment methods, including high-resolution air pollution models linked to maternal residential address, (interpolated) central air quality monitoring site data, and personal monitoring. As most important confounders we identified maternal age (prenatal studies) or child age (postnatal studies), maternal smoking, and an indicator of socio-economic status. All studies accounted for at least one, and most studies accounted for several of these potential confounders. No bias was introduced through the assessment of outcome, as brain imaging assessment was performed independent and blind. Follow-up was long enough for the outcome to occur, although brain development never truly stops. Adequacy of follow-up was a point of concern for many studies, mainly because of missing information about (selective) loss to follow up.

### 3.1 Fetal brain

Since January 2017, 11 studies investigating the association between *in utero* air pollution exposure and fetal brain measurements have been published: ten studies measured BPD and seven of them also HC (Table S3). One recent study used transvaginal neurosonography to acquire high-resolution images of the fetal central nervous system and reported fetal brain measures including the depth of the insula, Sylvian fissures, parieto–occipital, cingulate, and calcarine sulci, the width of anterior and posterior horns of lateral ventricles, third ventricle, and cisterna magna, and corpus callosum length, cerebellar transverse diameter, and vermis height (Gómez-Herrera et al., 2025).

Geographically, studies have broadened the scope and included samples acquired in countries with relatively high air pollution concentrations (namely China and South Korea) between 2017 and 2026, where previously studies were mainly conducted in countries with relatively low air pollution exposures (namely Europe, United States, and Australia) (Fu et al., 2019). For an overview, the findings have been visualized on a geographical world map (Fig. 2).

While some studies investigated exposure and outcome per trimester, others did not make this distinction. The studied pollutants include PM_2.5_, PM_10_, NO_2_, SO_2,_ O_3_, CO, and black carbon. The global prenatal brain measures BPD and HC generally appear to be inversely associated with prenatal exposure to air pollution (see Table S1 and Fig. 3 for an overview of directions of associations).

#### 3.1.1 PM_10_

Higher PM_10_ exposure was associated with smaller BPD in the third trimester but not in the second trimester in two studies (Lamichhane et al., 2018; Li et al., 2022) (Fig. 3). Associations for the third trimester did not remain significant when controlling for NO_2_ exposure (Lamichhane et al., 2018), while another study adjusting for NO_2_ and SO_2_ exposure observed a smaller BPD with higher PM_10_ exposure (Zhao et al., 2018). The same study reported a larger HC when exposure levels exceeded 150 μg/m^3^ (Zhao et al., 2018). The reported average exposure levels of these three studies were relatively high (83.6 μg/m^3^ (Li et al., 2022), 54.8 μg/m^3^ (Lamichhane et al., 2018) and in the most extreme case 140 μg/m^3^ (Zhao et al., 2018) compared to the study that did not report an association for HC and BPD (27.9 μg/m^3^) (Peterson et al., 2022a).

#### 3.1.2 PM_2.5_

Higher levels of PM_2.5_ were associated with a smaller BPD (Li et al., 2022; Cao et al., 2019), also when adjusting for SO_2_, NO_2_, PM_10_ and O_3_ (Cao et al., 2019). In a sub cohort of one of these studies (Cao et al., 2019), higher levels of PM_2.5_ were associated with a lower mean growth velocity per gestational week of HC and BPD (Cao et al., 2021). Another study found trimester-specific associations and reported smaller HC and BPD with higher PM_2.5_ exposure in the second trimester, but not in the third trimester (Zhao et al., 2021). One study investigating growth trajectories did not report an association between PM_2.5_ exposure and BPD growth (Chen et al., 2023). Another study showed that PM_2.5_ was associated with a wider anterior horn of lateral ventricles, wider cisterna magna, and larger cerebellar vermis, while no clear patterns or associations were found between air pollution and other structures of brain morphology (Gómez-Herrera et al., 2025). The reported average PM_2.5_ concentrations of the studies reporting associations with HC and BPD (Li et al., 2022; Cao et al., 2019, 2021; Zhao et al., 2021) were relatively high (∼40 μg/m^3^) compared to the studies that did not find an association for HC and BPD (11.7 μg/m^3^ (Peterson et al., 2022a) - 14.72 μg/m^3^ (Chen et al., 2023)) and the transvaginal neurosonography study (12.6 μg/m^3^) (Gómez-Herrera et al., 2025).

#### 3.1.3 NO_2_

NO_2_ exposure was associated with smaller BPD (Leung et al., 2023; Lamichhane et al., 2018; Wang et al., 2017) and HC (Zhao et al., 2018; Wang et al., 2017), although results varied between trimesters (Leung et al., 2023; Lamichhane et al., 2018) (Fig. 3). The findings did not differ when including PM_2.5_ (Leung et al., 2023) or PM_10_ (Lamichhane et al., 2018) in multi-pollutant models. One study also categorized exposure levels and observed smaller BPD in the high exposure group compared to the low exposure group (Wang et al., 2017). One study showed that NO_2_ was associated with a wider anterior horn of lateral ventricles, wider cisterna magna, and larger cerebellar vermis, while no clear pattern or associations were observed between air pollution and other structures of brain morphology (Gómez-Herrera et al., 2025). The reported average exposure levels of the three studies that reported negative associations were relatively high (46.9 μg/m^3^ (Lamichhane et al., 2018), 43.2 μg/m^3^ (Leung et al., 2023), 61.2 μg/m^3^ (Wang et al., 2017)) compared to the study that did not report an association for HC and BPD (15.5 ppb ≈ 29.1 μg/m^3^ (Peterson et al., 2022a)). The median exposure level of the transvaginal neurosonography study was 37.2 μg/m^3^ (Gómez-Herrera et al., 2025).

#### 3.1.4 O_3_

No significant associations were found between O_3_ exposure and HC or BPD with a median average exposure of (42.3 ppb ≈ 82.9 μg/m^3^) (Peterson et al., 2022a).

#### 3.1.5 SO_2_

Trimester-specific effects were reported for SO_2_, with exposure in the first trimester being associated with a smaller BPD in the third but not second trimester. Second trimester SO_2_ exposure was associated with smaller BPD in the third trimester (Li et al., 2022). Average exposure levels of around 11.9 μg/m^3^ were reported.

#### 3.1.6 CO

CO was investigated in the same study as SO_2_, and an association was reported between first trimester exposure and a smaller BPD in the third but not in the second trimester (Li et al., 2022). Average exposure levels of around 0.9 mg/m^3^ were reported.

#### 3.1.7 Black carbon

Black carbon was investigated in one study, in which black carbon exposure was associated with a wider anterior horn of lateral ventricles, wider cisterna magna, larger cerebellar vermis, and shallower Sylvian fissure in the third trimester (Gómez-Herrera et al., 2025). The median exposure level was 1.2 μg/m^3^.

### 3.2 Neonatal brain

Two MRI studies assessed associations between air pollution exposure *in utero* and brain structure in the neonatal period at a postmenstrual ages of 36.7-45.1 weeks (Bos et al., 2023) and 41.0-46.1 weeks (Pujol et al., 2025), respectively (Tables S2 and S4, Fig. 2, Fig. 3). Each of these studies focused on different regions of interests: One study focused on white matter, cortical gray matter, ventricles, extracerebral cerebrospinal fluid (CSF), cerebellum, hippocampus, amygdala, deep gray matter, and brainstem both with single-pollutant models and multi-pollutant CCA (Bos et al., 2023). In this study, single-pollutant models showed no significant associations while CCA reported correlations for global and specific brain regions. The other study investigated PM_2.5_ exposure and neonatal myelination as well as total brain volume and found higher exposure levels being associated with lower myelination (Pujol et al., 2025).

#### 3.2.1 PM_10_

Higher PM_10_ exposure was canonically correlated with larger ventricular, cerebellum, brain stem and extracerebral CSF, and smaller cortical gray matter, amygdala, and hippocampus volume. No associations were found for white matter and deep gray nuclei volume (Bos et al., 2023). The median PM_10_ concentration for the whole pregnancy was 23.4 μg/m^3^.

#### 3.2.2 PM_2.5_

No significant associations were reported between exposure to PM_2.5_ and local neonatal brain volumes with median PM_2.5_ levels of 13.5 μg/m^3^ (Bos et al., 2023), or total brain volume in the first or third trimester with mean exposure levels of 17.0 μg/m^3^ and 16.2 μg/m^3^, respectively (Pujol et al., 2025). Higher PM_2.5_ exposure in the first trimester of pregnancy was associated with lower cortical myelination, and higher exposure levels in the third trimester were associated with lower global myelination (Pujol et al., 2025).

#### 3.2.3 PM characteristics

Higher first trimester but not third trimester exposure to Fe and Cu was associated with lower cortical myelination in neonates (Pujol et al., 2025). However, associations did not remain significant when adjusting for PM_2.5_ exposure levels.

#### 3.2.4 NO_2_

Lower NO_2_ exposure was canonically correlated with larger ventricular, cerebellum, brain stem and extracerebral CSF, and smaller cortical gray matter, amygdala, and hippocampus volume. No associations were found for white matter and deep gray nuclei volume (Bos et al., 2023). The NO_2_ concentration during pregnancy had a median of 39.7 μg/m^3^.

### 3.3 Childhood and adolescent brain

A total of 13 studies investigated structural brain changes during late middle childhood (age range 4-17 years) using structural (n = 10) and/or diffusion MRI (n = 4) (Mortamais et al., 2019; Buthmann et al., 2025; Guxens et al., 2018; Kusters et al., 2024, 2025; Lewandowska et al., 2025; Lubczyńska et al., 2020, 2021; Margolis et al., 2022; Peterson et al., 2015, 2022b; Szwed et al., 2025; Yang et al., 2025), with two of the structural MRI studies and one of the diffusion MRI studies investigating longitudinal changes (Buthmann et al., 2025; Kusters et al., 2024, 2025) (Tables S2 and S4, Fig. 2, Fig. 3). The studied pollutants included PM_2.5_, PM_10_, PM_coarse_, NO_2_ and NO_X_. The characteristics of PM were also considered, with PM absorbance and PAHs being studied most frequently. Five studies were conducted within the Generation R cohort (Guxens et al., 2018; Kusters et al., 2024, 2025; Lubczyńska et al., 2020, 2021), two within the CCEH cohort (Peterson et al., 2015, 2022b), and two studies include the NeuroSmog cohort (Lewandowska et al., 2025; Szwed et al., 2025). In general, no significant associations were found for prenatal exposure to air pollution and global brain measures. However, changes in specific brain regions and white matter microstructures were reported (Table S4, Fig. 3).

#### 3.3.1 PM_10_

Six studies, four of which are part of the same cohort study, investigated PM_10_ exposure (Kusters et al., 2024, 2025; Lewandowska et al., 2025; Lubczyńska et al., 2020, 2021; Szwed et al., 2025). The structural MRI studies reported a larger cerebellum (Kusters et al., 2025; Lubczyńska et al., 2021) and faster growth of cerebellum and hippocampus (Kusters et al., 2025). One diffusion MRI study reported lower global fractional anisotropy (FA) and higher global mean diffusivity (MD) (Lubczyńska et al., 2020), while another reported no association for FA or MD or fixel-based analysis (FBA) (Lewandowska et al., 2025). However, PM_10_ was not identified as a predictor for cerebellum volume, FA, or MD in the multi-pollutant approach using the DSA algorithm (Lubczyńska et al., 2020, 2021). Lower global FA persisted at older ages, while no association was found for MD (Kusters et al., 2024). No associations with cortical myelin content were reported except for lower myelin content in the left precuneus (Szwed et al., 2025). The average exposure ranged from ∼27 μg/m^3^ (Kusters et al., 2024, 2025; Lubczyńska et al., 2020, 2021) to ∼45 μg/m^3^ (Lewandowska et al., 2025; Szwed et al., 2025).

#### 3.3.2 PM_coarse_

Exposure to PM_coarse_ was investigated in five studies within the same cohort (Guxens et al., 2018; Kusters et al., 2024, 2025; Lubczyńska et al., 2020, 2021). Diffusion MRI studies did not find any cross-sectional and longitudinal associations with global FA and global MD (Kusters et al., 2024; Lubczyńska et al., 2020). One structural MRI study reported thinner cortices in the right lateral orbitofrontal region (Guxens et al., 2018). Two studies reported a larger cerebellum (Kusters et al., 2025; Lubczyńska et al., 2021), one of which also found a larger putamen and pallidum (Lubczyńska et al., 2021) and the other a larger corpus callosum and a faster decline in total white matter across childhood and adolescence (Kusters et al., 2025). With multi-pollutant approaches, the association with cerebellum volume remained consistent in one study that applied the DSA algorithm (Lubczyńska et al., 2021) but not in the study that applied LASSO (Kusters et al., 2025). All studies had similar PM_coarse_ concentrations (median 9.9—11.8 μg/m^3^).

#### 3.3.3 PM_2.5_

Nine studies, including five studies from the same cohort, investigated changes in structural brain measures (Mortamais et al., 2019; Buthmann et al., 2025; Guxens et al., 2018; Kusters et al., 2024, 2025; Lubczyńska et al., 2020, 2021; Peterson et al., 2022b; Szwed et al., 2025). Larger cerebellum volume (Kusters et al., 2025; Lubczyńska et al., 2021) and reduced cortical thickness in the right hemisphere of the precuneus, pars opercularis, pars orbitalis, rostral middle frontal and superior frontal regions, and in the left hemisphere of the cuneus region (Guxens et al., 2018) were found. One study reported faster growth of hippocampal volume and cerebellum (Kusters et al., 2025), whereas in a younger sample, associations with hippocampal growth differed across the hemisphere and specific exposure window (Buthmann et al., 2025). One study reported a smaller corpus callosum but findings did not remain significant with false discovery rate correction (Mortamais et al., 2019). While higher global MD and lower global FA were observed in one study (Lubczyńska et al., 2020), another reported a higher regional FA (Peterson et al., 2022b). In a multipollutant analysis using the DSA algorithm, the association with cerebellar volume was no longer significant (Lubczyńska et al., 2021), but the association with lower global FA remained significant (Lubczyńska et al., 2020). Results for FA remained consistent over age in the longitudinal study, both in single- and multi-pollutant models, while no association for MD was found across ages (Kusters et al., 2024). One study reported varying results, with both increasing and decreasing white matter volumes and cortical thickness (Peterson et al., 2022b). Adjusting for PAH in the sensitivity analysis did not alter the results (Peterson et al., 2022b). PM_2.5_ exposure was not associated with cortical myelin content (Szwed et al., 2025). Median exposure levels ranged from 11.06 μg/m^3^ (Szwed et al., 2025) to 23.6 μg/m^3^ (Mortamais et al., 2019).

#### 3.3.4 PM characteristics

Few studies investigated PM content and (chemical) characteristics rather than solely classifying PM by size (Guxens et al., 2018; Lubczyńska et al., 2020, 2021; Margolis et al., 2022; Peterson et al., 2015, 2022b). PAH was associated with regional volume changes (Kusters et al., 2025; Lubczyńska et al., 2021; Peterson et al., 2015, 2022b) and higher regional FA (Peterson et al., 2022b). One study only showed significantly lower global FA with a multi-pollutant approach (Lubczyńska et al., 2020). Another study reported no direct effects of PAH, but *in utero* PAH exposure did moderate the association between maternal perceived stress assessed when children were 5 years old and regional brain volumes between ages 7 and 9 years (Margolis et al., 2022). Further, PAH-DNA adducts in maternal blood during pregnancy were associated with smaller hippocampal volumes in adolescence (Yang et al., 2025). PM_2.5_ absorbance was associated with a thinner cortex in the left fusiform region (Guxens et al., 2018) and the rostral middle frontal gyrus of the right hemisphere (Lubczyńska et al., 2021), larger cerebellum (Kusters et al., 2025; Lubczyńska et al., 2021), smaller corpus callosum (Kusters et al., 2025), and faster hippocampal and cerebellum growth (Kusters et al., 2025), lower global FA cross-sectionally (Lubczyńska et al., 2020) and longitudinally (Kusters et al., 2024), and higher global MD (Lubczyńska et al., 2020). Individual studies also detected structural changes in relation to Zn, Fe, Cu, Si, Fe, B[a]P, OC, OP_ESR_, OP_DTT_ and UFP exposure (Kusters et al., 2025; Lubczyńska et al., 2020, 2021). While some results stayed consistent when applying the DSA algorithm (Lubczyńska et al., 2020, 2021) or LASSO (Kusters et al., 2024, 2025), other associations were no longer detectable or emerged (Kusters et al., 2024, 2025; Lubczyńska et al., 2020, 2021), highlighting the difficulty of handling multiple correlated exposures.

#### 3.3.5 NO_2_/NO_x_

NO_2_ and NO_x_ were investigated in seven studies (four from the same cohort) (Guxens et al., 2018; Kusters et al., 2024, 2025; Lewandowska et al., 2025; Lubczyńska et al., 2020, 2021; Szwed et al., 2025). In one of the structural MRI studies, higher prenatal exposure to NO_2_ was associated with a smaller corpus callosum and faster cerebellum growth, and NO_x_ exposure was associated with a smaller a cerebellum and corpus callosum in adolescence (Kusters et al., 2025). No other associations of NO_2_ with regional brain volumes (Guxens et al., 2018; Kusters et al., 2025; Lubczyńska et al., 2021) and cortical thickness (Guxens et al., 2018) were found. One study investigating both NO_2_ and NO_x_ reported that higher levels of both pollutants were associated with higher global MD, and that higher exposure to NO_x_ was associated with lower global FA (Lubczyńska et al., 2020). These associations did not remain significant when applying the DSA algorithm (Lubczyńska et al., 2020). In the longitudinal study of the same cohort, associations with both NO_2_ and NO_x_ were not significant for MD and FA (Kusters et al., 2024), and in another study, NO_2_ was not associated with FA, MD, and FBA (Lewandowska et al., 2025). No associations were found between prenatal exposure to NO_2_ and cortical myelin content (Szwed et al., 2025). The median concentrations of NO_2_ in these studies ranged from 34.1 μg/m^3^ (Kusters et al., 2025) to 26.96 ppb (≈ 49.6 μg/m^3^ (Szwed et al., 2025)). For NO_x_ the median concentrations were between 46.4 and 50.8 μg/m^3^ (Kusters et al., 2025; Lubczyńska et al., 2020, 2021).

## 4 Discussion

We reviewed neuroimaging evidence linking *in utero* air pollution exposure with brain structure from the fetal through adolescent period, focusing on studies investigating associations with *in utero* exposure to the most commonly investigated air pollutants. Multiple reviewed studies reported several associations between *in utero* air pollution exposure and alterations in brain size; however, they revealed no evident pattern distinguishing effects by individual pollutant. Prenatal ultrasound studies showed a negative relationship between global brain structures, and *in utero* air pollution exposure, especially in high-pollution areas. In contrast, MRI studies of children and adolescents, predominantly from lower-exposure areas, reported no significant associations between overall brain volumes and *in utero* air pollution exposure, except for lower global white matter microstructure. Although most local brain structures in children and adolescents were not implicated, a larger cerebellum in two studies and a smaller corpus callosum in one study were associated with *in utero* air pollution exposure.

Studies on prenatal brain development demonstrated associations between *in utero* air pollution exposure and global fetal brain growth, with smaller HC and/or BPD across pollutant types and trimesters (Leung et al., 2023; Lamichhane et al., 2018; Li et al., 2022; Zhao et al., 2018, 2021; Cao et al., 2019, 2021; Chen et al., 2023; Wang et al., 2017). Unlike earlier research (Fu et al., 2019), studies in this review were primarily conducted in higher-pollution areas. Integrating and extending these findings with a meta-analysis performed in 2019 (Fu et al., 2019), associations between global fetal brain structures and *in utero* air pollution exposure were more evident in high-pollution regions (Leung et al., 2023; Lamichhane et al., 2018; Li et al., 2022; Zhao et al., 2018, 2021; Cao et al., 2019, 2021; Wang et al., 2017), compared to low-exposure settings (Fu et al., 2019; Peterson et al., 2022a). That meta-analysis yielded no significant links between fetal HC and NO_2_ or PM_10_, reporting effect sizes of −0.02 (−0.12, 0.07) for NO_2_ and –0.12 (−0.28, 0.04) for PM_10_ per 10 μg/m^3^ increase in pollutant concentration, and their qualitative synthesis remained inconclusive (Fu et al., 2019). Arguably, in low-exposure regions, effects may be too subtle for detection with 2D ultrasound measures like HC and BPD, which serve as indirect proxies for global brain volumes. However, a recent transvaginal 2D neurosonography study did look beyond HC and BPD and indicated structural changes of smaller subregions in the prenatal brain (Gómez-Herrera et al., 2025). While alternatively, air pollution might only affect fetal brain above a critical threshold, as evidenced by a reported (non-linear) relationship between NO_2_ exposure and smaller HC at birth (Ballester et al., 2010), for many outcomes there is evidence that there are air pollution effects at very low concentrations (World Health Organization, 2021).

Postnatal neuroimaging studies revealed some associations between *in utero* air pollution exposure and brain structural differences in infancy, childhood, and adolescence. No significant effects on global measures, such as total brain volume, overall gray matter, and white matter volumes, ​were reported (Mortamais et al., 2019; Guxens et al., 2018; Lubczyńska et al., 2021)​, except for global FA (Kusters et al., 2024; Lubczyńska et al., 2020). Regional analyses showed mixed results with PM exposure: positive associations for cerebellum, putamen, pallidum, and amygdala volumes; negative associations for certain regional cortical thickness areas, hippocampus and corpus callosum volumes, and for several white matter surface areas (Guxens et al., 2018; Lubczyńska et al., 2021; Peterson et al., 2022b). In addition, global white matter microstructure alterations were associated with *in utero* air pollution exposure, including lower global FA (Kusters et al., 2024; Lubczyńska et al., 2020) and higher subcortical FA (Peterson et al., 2022b). White matter integrity, measured by FA, typically increases throughout childhood and adolescence, and these developmental increases are positively associated with cognition and behavior (Koenis et al., 2018; Michel et al., 2024). Stunted white matter development originating in early life may thus lead to functional alterations. While research on prenatal air pollution exposure and axon myelination is still limited, two studies indicated an association of higher air pollution with lower myelin content in the brain (Pujol et al., 2025; Szwed et al., 2025). Myelination is critical for increasing neural signaling speed and thus for complex information processing and psychological functioning (Baum et al., 2022). Safeguarding fetal brain development from air pollution could therefore benefit both short-term and long-term outcomes.

Directly linking *in utero* air pollution exposure to brain structure in childhood and adolescence remains challenging due to potentially unaccounted confounding factors, gene-by-exposure interactions, and postnatal exposure from birth until MRI (usually late middle childhood). The exposome framework addresses this by encompassing multiple exposures, such as synthetic chemicals, dietary constituents, psychosocial stressors, and physical factors, across the lifespan (Vermeulen et al., 2020). Comprehensive exposome-by-genome assessments could aid in clarifying neurodevelopmental variability (Liu et al., 2023). As neurodevelopment extends beyond childhood and adolescence, exposure to air pollution during childhood and adolescence may also impact brain development with implications for cognition and mental health (Herting et al., 2024). Effects might accumulate over time, with effects of postnatal exposure becoming more pronounced in older children and adolescents. A study, not discussed in this review due to a continued exposure window reaching into childhood, used distributed lag non-linear models to explore susceptibility windows between conception and time of MRI scan. They found key susceptibility windows to NO_2_, PM_2.5_ and PM_2.5_ absorbance from conception to age 5 years in association with lower FA, higher MD and larger putamen volumes (Binter et al., 2022), highlighting the importance of early postnatal exposure in addition to *in utero* exposure. Sensitive windows of exposure varied per pollutant (lower FA (NO_2_: ages 3.6-4.8 years; PM_2.5_: conception-3.9 years; PM_2.5_ absorbance: conception-5 years), higher MD (NO_2_: 2.2-4.7 years; PM_2.5_: 9 months-4.4 years; PM_2.5_ absorbance: 1.1-4.8 years), and a larger putamen volume (PM_2.5_: 4 months–1.8 years)) (Binter et al., 2022). Of the studies that we reviewed here, some investigated not only *in utero* exposure but also postnatal exposure windows in relation to brain development (Mortamais et al., 2019; Kusters et al., 2024, 2025; Lewandowska et al., 2025; Lubczyńska et al., 2020, 2021). However, pre- and postnatal exposures are usually correlated (Pearson's correlations coefficients in these studies ranged from 0.47 to 0.81 (Mortamais et al., 2019; Kusters et al., 2025; Kusters et al., 2024; Lewandowska et al., 2025; Lubczyńska et al., 2021; Lubczyńska et al., 2020)) which makes it challenging to disentangle the importance of pre- and postnatal exposure. A few studies reported similar associations between air pollution exposure and brain outcomes across pre- and postnatal exposure windows (Kusters et al., 2024, 2025; Lubczyńska et al., 2020, 2021), while others only reported effects of *in utero* exposure (Mortamais et al., 2019) or postnatal exposure (Lewandowska et al., 2025). Thus, both periods may be potentially impactful, and the specific role of *in utero* exposure for long-term effects is still inconclusive.

While evidence is very limited, sensitive periods might vary between volumetric and microstructural brain outcomes. One study on volumetric brain measures reported effects only for *in utero* exposure that remained stable when correcting for current exposure, but no effects were found for exposure between age 0 and 2 years (Mortamais et al., 2019). In another study on white matter integrity, no effects of *in utero* exposure were observed, whereas FA and MD were associated with exposure between age 0 and 4 years (Mortamais et al., 2019). Different windows of susceptibility between pollutants and brain regions (Lewandowska et al., 2025), suggest that specific developmental processes as well as characteristics of individual pollutants (e.g., ability to cross the placenta) are playing a role. However, reliance on single MRI timepoints limits insight. Longitudinal designs with repeated scans and accurate assessment of (continuous) lifetime exposures would better reveal air pollution's cumulative effects, given varying regional vulnerabilities over development.

Geographical differences in pollution levels were observed. For instance, the world map (Fig. 2) provides an overview of how PM_2.5_ levels differ between countries and continents. Studies assessing influences of *in utero* air pollution exposure on fetal and postnatal brain development are unevenly distributed: lower pollution areas are underrepresented in fetal ultrasound research, while higher-pollution regions are underrepresented in infant, child, and adolescent MRI studies. This imbalance makes the interpretation of findings preliminary and challenging. Nonetheless, these patterns justify further investigation into air pollution particles’ impact on brain development. Identifying particularly impactful particles from specific sources could guide targeted environmental interventions.

Evidence for pollutant-specific effects is currently insufficient. Depending on the presence of specific sources and the pollutant mixture (e.g., particle size distributions and composition), and consequently biological activity, effects can differ between brain regions. Smaller particles are potentially more harmful due to their larger surface area/mass ratio, enhanced oxidative capacity, and ability to translocate into systemic circulation (Künzli et al., 2010; Johnson et al., 2021). However, imaging studies do not allow a conclusion based on size, since effects of *in utero* air pollution exposure on the brain were reported for PM_2.5_, PM_coarse_ and PM_10_. PM_10_ overlaps with PM_2.5_ and with PM_coarse_ and exposure levels are often highly correlated because of shared sources. Moreover, UFPs have rarely been investigated due to a lack of routine monitoring and fine scale models (Costa et al., 2020; Johnson et al., 2021). Childhood studies implicate that PM composition and characteristics, such as PAH (Lubczyńska et al., 2020, 2021; Peterson et al., 2022b; Yang et al., 2025), oxidative potential, and absorbance (Kusters et al., 2024, 2025; Lubczyńska et al., 2020, 2021) may play a role for potential effects of PM. Gaseous pollutants (SO_2_, O_3_, CO, and NO_x_) and black carbon have been sparsely investigated. For some pollutants, concentrations decreased significantly in recent years, which has been achieved by implementing various measures (i.e., fuel switching from high sulfur solids (e.g., coal) to low sulfur fuels (e.g., natural gas) in energy-related sectors) (European Environment Agency (EEA), 2013). However, with road traffic being a major source of CO and NO_x_, and O_3_ forming through reactions between NO_x_ and volatile organic compounds (Künzli et al., 2010), there is a need for further research.

Prenatal air pollution has been associated with a wide range of adverse effects on neurodevelopment and long-term consequences impacting cognition and behavior (Yi et al., 2022). Elevated NO_2_ associates with autism spectrum disorder incidence (Volk et al., 2013), lower verbal development at age 7 (Porta et al., 2016), increased inattentiveness at ages 4-5 (Sentís et al., 2017), behavioral problems at ages 2-4 (Ni et al., 2022), and cognitive and adaptive deficits in a sample of children with autism spectrum disorder (Kerin et al., 2018). Similarly, high levels of PM were associated with autism spectrum disorder incidence (Volk et al., 2013), PAH exposure with lower IQ at age 5 (Edwards et al., 2010; Perera et al., 2009), third-trimester CO with lower neuropsychological performance at ages 6-7 (Dix-Cooper et al., 2012), and overall air pollution with impaired cognitive development through age 7 (Gonzalez-Casanova et al., 2018). To elucidate underlying pathways, integrated studies of brain structure and cognition in pollution context are essential. *In utero* PM exposure partially mediated inhibitory control (ages 6-10) via reduced precuneus and rostral middle frontal cortex volume (Guxens et al., 2018). Prenatal air pollution exposure has been associated with smaller body corpus collosum volume and was linked to hyperactivity by ages 8-12 years (Mortamais et al., 2019). One study indicates that prenatal PAH exposure affects hippocampal volume, subsequently leading to worse reading ability in adolescence (Yang et al., 2025). There is ample evidence for brain structures to be associated with cognition (Koenis et al., 2018; Schnack et al., 2015; Shaw et al., 2006) and behavior (Teeuw et al., 2023) during childhood and adolescence. Moreover, there is increasing evidence for a long-term impact of brain health on mental health based on longitudinal studies (Hulshoff Pol and Brouwer, 2025) which could potentially inform personalized medicine approaches in the future (Bozek et al., 2023; Chamberland et al., 2021; Ge et al., 2024). Thus, although evidence of *in utero* air pollution exposure on childhood and adolescent brain development is mixed, it suggests that long-lasting effects of *in utero* air pollution exposure may be present, either direct or mediated via structural brain changes. It underlines the need for further investigation as even small changes in cognitive development or social performance can have considerable consequences (Künzli et al., 2010; Rice and Barone, 2000).

Several limitations have to be taken into account when interpreting the results of this literature review. The heterogeneity of the study samples, exposure levels across geographic regions, and methodological approaches (e.g., exposure assessment and statistical analysis) used to assess the impact of air pollution on brain development differed greatly between studies and therefore limits the comparability of studies. For instance, some quantified exposure levels of air pollution representing the entire pregnancy (Bos et al., 2023; Gómez-Herrera et al., 2025; Zhao et al., 2018, 2021; Peterson et al., 2015, 2022a, 2022b; Cao et al., 2019, 2021; Guxens et al., 2018; Kusters et al., 2024, 2025; Lubczyńska et al., 2020, 2021; Margolis et al., 2022; Szwed et al., 2025), while others investigated trimester-specific exposures (Lamichhane et al., 2018; Li et al., 2022; Wang et al., 2017; Pujol et al., 2025; Chen et al., 2020). Also, exposure assessment differed between studies. Most studies included in this review used fine-scale air pollution modelling, more specifically stochastic land-use regression (LUR) modelling that combines monitoring data to develop empirical models using traffic, population and land use predictor variables (Jerrett et al., 2005). LUR models differed with regard to the type of monitoring data (e.g., routine monitoring at fixed monitoring sites, mobile monitoring and satellite remote sensing data) and the model development algorithms used (e.g., methods, including linear regression and increasingly machine learning methods). A recent systematic review suggested that machine-learning methods may be superior to linear non-regularized statistical methods in predicting ambient concentrations of NO_2_, UFPs and black carbon, but differences in performance between these algorithms were often small and varied between studies (Hoek, 2017). Comparisons of associations with health outcomes (e.g., childhood asthma and lung function, mortality) suggested generally consistent results with regard to the presence and direction of the associations but difference in magnitude (Bouma et al., 2025a, 2025b). Such comparisons are not available for prenatal air pollution exposure and brain development. Air pollution models were linked to the participants’ residential addresses, not taking into account time-activity patterns and exposure at other locations assuming that participants, and children in particular, spend the largest part of their time at home. A recent review of exposure studies that have compared correlations and health effect estimates between residential and time-activity integrated exposures concluded that bias related to assessing long-term exposure at the residential address only is likely small (Hoek et al., 2024). Few studies included in this review relied on personal monitoring (Zhao et al., 2021; Peterson et al., 2015, 2022b), which is a direct method that has the advantage that it accounts for exposures at locations other than home, but is limited to shorter measurement durations (i.e., a few days or weeks) and is not feasible for large-scale studies.

Most studies relied on a limited set of exposures to represent the complex air pollution mixture. Also, in studies where multiple air pollutants have been assessed, analyses often relied on single-pollutant models, neglecting confounding of other (often highly correlated) pollutants and interactions between pollutants thereby limiting conclusions regarding pollutant-specific effects and consequently the relevant sources that should be addressed by policies. The advances towards multi-pollutant models have been encouraged by several policymakers and organizations to improve estimation of health risk and set regulations for multiple pollutants (Dominici et al., 2010). Efforts have been made towards multiple pollutant models in which effects of usually two or three exposures were mutually adjusted to assess pollutant-specific effects. These sets of multiple pollutants included all possible combinations in some studies (Lamichhane et al., 2018; Zhao et al., 2018) or combinations of pollutants selected by different algorithms, e.g., the LASSO (Kusters et al., 2024, 2025) or the DSA algorithm (Lubczyńska et al., 2020, 2021) that compromises sensitivity and false discovery rate. Multicollinearity is a major challenge of conventional multi pollutant models if pollutants are highly correlated (Dominici et al., 2010). Other multiple regression methods to approach the air pollution mixture include LASSO, ridge regression and elastic net (Agier et al., 2016), which were found to outperform conventional approaches. Of these, LASSO and ridge regression have been used in the reviewed studies (Gómez-Herrera et al., 2025; Kusters et al., 2024, 2025). A limitation of using LASSO for assessing pollutant-specific effects is that it randomly selects variables if variables are highly correlated (Tibshirani, 1996), while ridge regression might keep uninformative predictors in the model. One study used CCA to assess associations between multiple correlated exposures and multiple brain volume outcomes simultaneously (Bos et al., 2023).

Sample sizes in the reviewed studies varied widely. The number of included participants ranged from 37 to 8877 participants per study, considerably affecting the power to detect smaller subtle effects within the brain. Moreover, ultrasound studies routinely measured HC and/or BPD via 2D scans as fetal growth proxies. However, these can overlook subtle anatomical changes within the brain. One recent study used 2D transvaginal rather than more commonly used transabdominal neurosonography and found more subtle differences in cortical folding patterns, in CSF spaces, and cerebellar vermis (Gómez-Herrera et al., 2025). In addition, considering that postnatal MRI studies detected regional alterations without impacting global brain measures, one could argue that similarly, smaller, undetectable prenatal effects might have been overlooked when only investigating global metrics as HC and BPD. Besides advances in transvaginal ultrasound, also fetal 3D ultrasound and MRI promise much finer assessment of the developing prenatal brain (Bastiaansen et al., 2023; Lautarescu et al., 2024; Merz and Pashaj, 2017; Namburete et al., 2023; de Zwarte et al., 2024). Recently, efforts have been made to create a normative fetal brain atlas, that could support more detailed investigations of fetal brain maturation, including finer anatomical details (Namburete et al., 2023). In the studies that investigated prenatal air pollution exposure and postnatal MRI measures, a variety of brain regions of interest were investigated, e.g., gray or white matter volume (Mortamais et al., 2019; Bos et al., 2023; Guxens et al., 2018; Lubczyńska et al., 2021; Margolis et al., 2022; Peterson et al., 2022b), cortical thickness (Guxens et al., 2018; Lubczyńska et al., 2021; Peterson et al., 2022b), surface morphology (Peterson et al., 2015), or white matter integrity (Kusters et al., 2024; Lubczyńska et al., 2020). While these heterogeneous studies give a more detailed insight into brain development, a direct comparison is limited. The MRI studies were also primarily conducted in areas with low levels of air pollution exposure; therefore, it remains unclear to what degree these findings are representative to the pollution exposure levels of the global population. Only two MRI studies investigated neonatal brain development in the context of air pollution (Bos et al., 2023; Pujol et al., 2025) and only one other study included children below the age of 6 (Buthmann et al., 2025). To bridge the gap and to link prenatal to postnatal development, study designs in which children are followed from the prenatal period throughout their development are essential. In addition, it is also key to assess lifetime exposures to air pollution with high temporal resolution to disentangle the importance of exposures during different time periods. With promising developments in the field of prenatal neuroimaging and automated processing, there are now opportunities to not only map global but also regional brain measures. This will allow not only for monitoring global brain development from the prenatal period throughout life but, equally as important, also for regional brain development. These future longitudinal studies could provide a better insight in the developmental processes and the relation of short- and longer-term effects.

To conclude, we discussed recent research on *in utero* air pollution exposure and its effects on brain development from the prenatal period to adolescence. Convergent evidence so far suggests that *in utero* air pollution exposure can influence the developing fetal central nervous system when the air pollution is severe irrespective of the particles present. Prenatal ultrasound studies reported negative associations between *in utero* air pollution exposure and global measures of fetal brain size, suggesting restricted overall brain growth with higher exposure. These global associations, amplified at higher pollutant concentrations were not reproduced in postnatal MRI studies, which were primarily based in lower-exposure regions. Instead, MRI findings pointed to more localized alterations in specific brain regions, white matter microstructure and myelination, changes that may underlie later cognitive and behavioral outcomes despite preserved global volumes. This methodological and geographic divide implies that prenatal ultrasound may be sensitive to gross growth alterations apparent only under high exposure conditions, whereas MRI captures subtler, region-specific neurodevelopmental effects more typical of lower exposures. The uneven global distribution of studies complicates direct comparison and highlights the need for geographically diverse, longitudinal imaging designs combining global and local brain metrics to better characterize *in utero* air pollution's effects on neurodevelopment. Ultimately, a better understanding of long-term effects and underlying processes may be crucial to improve brain health and overall public health.

## Declaration of competing interest

The authors declare that they have no known competing financial interests or personal relationships that could have appeared to influence the work reported in this paper.
