# Supplementary material for: In utero air pollution exposure and pre- and postnatal brain development: a review
Source: Neurosci Appl. 2026 Jul 11;5:107019. doi: 10.1016/j.nsa.2026.107019 (PMC13393808; doi:10.1016/j.nsa.2026.107019)
Supplement: Multimedia component 1 [file mmc1.docx]

**Supplementary tables: In-utero air pollution exposure and pre- and postnatal brain development: a review**

Vera Goossens^a^*, Nora L. Großmann^b^*, Ulrike Gehring^c^, Mireille N. Bekker^d^, Hilleke E. Hulshoff Pol^a^, Sonja M.C. de Zwarte^b,e^

^a^ Department of Experimental Psychology, Helmholtz Institute, Utrecht University, Utrecht, The Netherlands

^b^ Department of Developmental Psychology, Utrecht University, Utrecht, The Netherlands

^c^ Institute for Risk Assessment Sciences, Utrecht University, Utrecht, The Netherlands

^d^ Department of Obstetrics, University Medical Center Utrecht, Utrecht, The Netherlands

^e^ Department of Psychiatry, UMC Brain Center, University Medical Center Utrecht, Utrecht University, Utrecht, The
 Netherlands

* These authors contributed equally to this work.

**Table S1**. Directions and statistical significance of associations between *in utero* air pollution exposure and fetal brain development in ultrasound studies published between January 1, 2017 and February 25, 2026. Reference numbers are provided in brackets.

| **Brain measurement** |  | **PM_10_** | **PM_2.5_** | **NO_2_** |
| --- | --- | --- | --- | --- |
| HC | 2^nd^ trimester |  | ↓ (1) | ↓ (2)​ ↓ (3)​* |
|  | 3^rd^ trimester | ↑ ​(4) | ↑ ​(1)​ ­↓ ​(4)​ | ↓ ​(2) ​ ↓ ​(3)​** ↓ ​(4)​ |
|  | Repeated measures |  | ↓ (1) | ↓ ​(2) ​ |
| BPD | 2^nd^ trimester | ↓ ​(5)​ ↓​ (6) ​ | ↓ ​(1)​  ↓ ​(5) ​ ­↓ (7)​ ​*** | ↑​(2) ​ ↓ ​(3)​* ↓ ​ (6) ​ |
|  | 3^rd^ trimester | ↓ ​(4)​ ↓ ​(5) ​ ↓ ​(6) | ↑ ​(1)​ ↓ ​(4)​ ↓ ​(5) ​ ↓​ (7)​*** | ↓ ​(2) ​ ↓ ​(3)​  ↑​(4)​ ↓ ​ (6) ​ |
|  | Repeated measures |  | ↓ ​(1)​ ↓ ​(8) | ↓ ​​(2) |

Direction and statistical significance of association of particulate matter with a diameter ≤ 10 μm (PM_10_), particulate matter with a diameter ≤ 2.5 μm (PM_2.5_), and nitrogen dioxide (NO_2_) with prenatal head circumference (HC) and biparietal diameter (BPD): arrows indicate that higher exposure is associated with lower (↓ ) or higher values (↑). Significant associations (p-value < 0.05) are presented in red, non-significant associations are presented in black. Representation of findings are simplified and might not always represent multiple analyses within one study.

* Only statistically significant with cumulative exposure but not semester specific exposure, gestational age: 24-31 weeks; ** Only statistically significant with Trimester 2 exposure but not cumulative exposure, gestational age: 32-40 weeks; *** Depending on exposure window.

Not including studies that reported only multi-pollutant models (Zhao et al., 2018), growth velocity (Cao et al. 2019), or brain measures other than BPD and HC (Gómez-Herrera et al. 2025).

**Table S2**. Directions and statistical significance of the associations between brain measurements (neonatal, childhood, adolescence) and *in utero* exposure to most frequently studied air pollutants. Structural neuroimaging studies in children and adolescents aged 0-17 years and published between January 1, 2000, and February 25, 2026 were included. Reference numbers are provided in brackets.

|  | **NO_2_/NO_x_** | **PM_10_** | **PM_coarse_** | **PM_2.5_** |
| --- | --- | --- | --- | --- |
| Total brain volume | ↑​(9)​↑​(10) | ↑​(10)​ | ↓​(9)​↑​(10)​ | ↓​(9)​↑​(10)​↓​(11)​ |
| Lateral ventricles volume | ↑​(9)​↓​​(12) | ↑​(12)​ | ↓​(9)​ | ↓​(9)​↑(13)​↓​(12)​ |
| Total white matter volume | ↑(10)↓(12)↑(14) | ↑(10)↓(12)↑(14) | ↑(10)↑(14) | ↑(10)↓(12)↑(14)↓(13) |
| Cortical white matter volume | ↑(9)↑(14) | ↑(14) | ↓(9)↑(14) | ↓(9)↑(14) |
| Local white matter volume |  |  |  | ↓↑(15)* |
| Cortical gray matter volume | ↓​(9)​↑​(10)↑(12)↑(14) | ↑​(10)↓(12)↑(14) | ↓(9)↑(14) ↑(10) | ↓​(9)​↑​(10)↑(12)↑(14) |
| Subcortical gray matter volume | ↑​(9)​↑​(10) | ↑​(10) | ↑​(9)​ ↑(10) | ↑​(9)​↑​(10)}↑(13) |
| Cortical thickness of subregions |  |  | ↓​(9) | ↓​(9) |
| Corpus callosum volume | ↓(10)↓(14) | ↓(10)↓(14) | ↑(10)↑(14) | ↑(10)↓(13)↓(14) |
| Cerebellum volume | ↑(10)↑(14)↓(12) | ↑(10)↑(14)↑(12) | ↑(10)↑(14) | ↑(10)↑(14)↑(12) |
| Thalamus volume | ↑↓(10)↓(14) | ↑(10)↑(14) | ↑(10)↑(14) | ↑(10)↑(14) |
| Caudate nucleus volume | ↑(10)↑(14) | ↑(10)↑(14) | ↑(10)↑(14) | ↑(10)↑(14) |
| Putamen volume | ↑(10)↑(14) | ↑(10)↑(14) | ↑(10)↑(14) | ↑(10)↑(14) |
| Pallidum volume | ↑↓(10)↑↓(14) | ↓(10)↑(14) | ↑(10)↑(14) | ↑(10)↑(14) |
| Hippocampus volume | ↓(10)↓(14)↓(12) | ↓(10)↓(14)↓(12) | ↓(10)↑(14) | ↓(10)↓(14)↓(12) |
| Amygdala volume | ↓(10)↓(14)↓(12) | ↓(10)↓(14)↓(12) | ↓(10)↑(14) | ↓(10)↓(14)↓(12) |
| Nucleus accumbens volume | ↑(10)↑(14) | ↑(10)↑(14) | ↑(10)↑(14) | ↑(10)↑(14) |
| Deep grey nuclei volume | ↓(12) | ↑(12) |  | ↑(12) |
| Brainstem volume | ↓(12) | ↑(12) |  | ↓(12) |
| CSF (extra cerebral) volume | ↑(12) | ↑(12) |  | ↑(12) |
| Global FA | ↓(16)↓(17)↓(18) | ↓(16)↑(17)↓(18) | ↓(16)↓(18) | ↓(16)↓(18) |
| Local FA |  |  |  | ↑(15) |
| Global MD | ↑(16)↑(17)↑(18) | ↑(16)↓(17)↑(18) | ↑(16)↑(18) | ↑(16)↑(18) |
| Global myelination |  |  |  | ↓​(11)​** |
| Cortical myelination |  |  |  | ↓​(11)​*** ↓↑ (22)* |

Direction and statistical significance of association of particulate matter with a diameter ≤ 10 μm (PM_10_), particulate matter with a diameter ≤ 2.5 μm (PM_2.5_), and nitrogen dioxide (NO_2_) with brain measures during childhood and adolescence: arrows indicate that higher exposure is associated with lower (↓) or higher values (↑). Significant associations (p-value < 0.05) are presented in red, non-significant associations are presented in black. Representation of findings are simplified and might not always represent multiple analyses within one study.

*Positive or negative association depending on specific brain region; **Only statistically significant for fetal exposure; ***Only statistically significant for embryonic exposure. CSF: cerebrospinal fluid

**Table S3.** The relationship between *in utero* air pollution exposure and fetal brain development in ultrasound studies published between January 1, 2017 and February 25, 2026. An overview of study characteristics and main outcomes by pollutant (PM_10_, PM_2.5_, NO_2_, BC, SO_2_, CO and O_3_) is provided. Studies are ordered by year of publication, newest studies first.

|  |  |  |  |  |  |  | **Effect estimates (95% CI) [increment]** | |
| --- | --- | --- | --- | --- | --- | --- | --- | --- |
| **Reference / Cohort** | **Sample size** | **Country** | **Study design** | **GA at outcome assessment** | **Exposure assessment** | **Exposure period** | **Single-pollutant model** | **Multi-pollutant model** |
| *PM_10_* |  |  |  |  |  |  |  |  |
| Li et al. 2022 (5) / MIH-Hefei | 7419 pregnancies | China | Prospective birth cohort | US1: 14–27 wks  US2: 28–40 wks | Monitoring station located within 3 km of the home address | Trimesters 1, 2 and 3 | **T1 exposure** [26.6 μg/m^3^]: BPD z-score US1: −0.028 (−0.091, 0.036), BPD z-score US2: −0.065 (−0.107, 0.022)  **T2 exposure** [24.5 μg/m^3^]: BPD z-score US2: 0.060 (−0.104, −0.016) |  |
| Peterson et al. 2022 (4) / MADRES | 281 pregnancies | USA | Prospective pregnancy cohort | 32±2 wks | Inverse-distance square weighting of routine monitoring data linked to residential histories | Conception to US | **Pregnancy average exposure** [7.1 μg/m^3^]:  BPD: −0.2 (−0.9, 0.6) mm,  HC: 0.3 (−2.0, 2.7) mm |  |
| Lamichhane et al. 2018 (6) / MOCEH | 426-461 pregnancies | South Korea | Prospective birth cohort study | US1: 12–27 wks US2: 28–40 wks | Residential address linked to land-use regression model | Each trimester and whole pregnancy | **T1 exposure** [10 µg/m^3^]: BPD US1: −0.10 (−0.34, 0.14) mm, BPD US2: 0.11 (−0.13, 0.36) mm  **T2 exposure** [10 µg/m^3^]: BPD US1: −0.18 (−0.43, 0.07) mm, BPD US2: −0.01 (−0.34, 0.33) mm  **T3 exposure** [10 µg/m^3^]: BPD US2: −0.31 (−0.59, −0.03) mm | Adjusted for NO_2_  **T1 exposure** [10 µg/m^3^]: BPD US1: 0.08 (−0.18, 0.35) mm, BPD US2: 0.20 (−0.10, 0.49) mm **T2 exposure** [10 µg/m^3^]: BPD US1: −0.01 (−0.29, 0.26) mm, BPD US2: 0.11 (−0.27, 0.48) mm   - **T3 exposure** [10 µg/m^3^]: BPD US2: −0.34 (−0.82, −0.13) mm |
| Zhao et al. 2018  (19)/ Lanzhou Birth Cohort Study | 8877 pregnancies | China | Prospective birth cohort | 39±2 wks | Inverse distance weighting of routine monitoring data linked to residential histories and work addresses | Conception to US;  one month, two weeks, and one week before ultrasound |  | - Adjusted for NO_2_ and SO_2_ - **Conception to US** [10 µg/m^3^]**:** BPD z-score: −0.018 (−0.030, −0.007), HC z-score: 0.011 (−0.006, 0.029) - **1 month before US** [10 µg/m^3^]: BPD z-score: −0.004 (−0.008, 0.000), HC z-score: 0.005 (0.000, 0.009) - **2 weeks before US** [10 µg/m^3^]: BPD z-score: −0.002 (−0.006, 0.002), HC z-score: 0.008 (0.001, 0.011) [10 µg/m^3^]   **1 week before US** [10 µg/m^3^]: BPD z-score: −0.002 (−0.006, 0.002), HC z-score: 0.004 (0.000, 0.009) [10 µg/m^3^] |

**Table S3.** (continued)

|  |  |  |  |  |  |  | **Effect estimates (95% CI) [increment]** | |
| --- | --- | --- | --- | --- | --- | --- | --- | --- |
| **Reference / Cohort** | **Sample size** | **Country** | **Study design** | **GA at outcome assessment** | **Exposure assessment** | **Exposure period** | **Single-pollutant model** | **Multi-pollutant model** |
| *PM_2.5_* |  |  |  |  |  |  |  |  |
| Gómez-Herrera et al. 2025 (20) / BiSC * | 754 pregnancies | Spain | Prospective birth cohort study | 222 ± 9 days | LUR, dispersion and hybrid models (main model) linked to home and work addresses and commuting routes | Conception to US | Wider anterior horn of lateral ventricles, wider cisterna magna, larger cerebellar vermis | Wider anterior horn of lateral ventricles (model including NO_2_ and BC) |
| Chen et al. 2023 (7) / INMA | 2328 pregnancies | Spain | Prospective birth cohort study | US1: 12 wks  US2: 20 wks  US3: 34 wks | Spatio-temporal random forest model with back-extrapolation linked to residential histories | Early: 0–11  Mid: 12–19  Late.: 20–33 | %change in fetal growth parameter [5µg/m^3^]  **Early exposure**: Early BPD growth: −0.44 (−0.91, 0.03), Mid BPD growth: 0.11 (−0.30, 0.51), Late BPD growth: −0.10 (−0.37, 0.17)  **Mid exposure**: Mid BPD growth: −0.17 (−0.58, 0.24), Late BPD growth: 0.17 (−0.14, 0.47)  **Late exposure**: Late BPD growth: −0.13 (−0.39, 0.13) |  |
| Li et al. 2022 (5) / MIH-Hefei | 7419 pregnancies | China | Prospective birth cohort | US1: 14–27 wks  US2: 28–40 wks | Monitoring station located within 3 km of the home address | Trimesters 1, 2 and 3 | **T1 exposure** [30.2 μg/m^3^]: BPD z-score US1: −0.089 (−0.155, −0.024), BPD z-score US2: −0.080 (−0.128, −0.031)  **T2 exposure** [27.8 μg/m^3^]: BPD z-score US2: −0.060 (−0.104, −0.016) |  |
| Peterson et al. 2022 (4) / MADRES | 281 pregnancies | USA | Prospective pregnancy cohort | 32±2 wks | Inverse-distance square weighting of routine monitoring data linked to residential histories | Conception to US | **Pregnancy average exposure** [2.2 μg/m^3^] BPD: −0.4 (−1.2, 0.4) mm,  HC: −1.0 (−3.5, 1.6) mm |  |
| Cao et al. 2021  (21) / SMILE | 6129 pregnancies | China | Prospective cohort | 14–41 wks; repeated measurements | Inverse-distance square weighting of routine monitoring data linked to residential addresses | Conception to the last week before US | **Cumulative exposure** [10 μg/m^3^]  BPD growth: −0.02 (−0.03, −0.01) mm/wk,  HC growth: −0.12 (−0.15, −0.09) mm/wk |  |
| Zhao et al. 2021  (1) / Shanghai MCPC | 287 pregnancies | China | Prospective cohort | US1: 21±2 wks,  US2: 32±1 wks | Random forest model based on personal monitoring and satellite data driven ambient concentrations | M1: conception to US1  M2: conception to US2  and entire pregnancy | **Second trimester** [1 μg/m^3^]: BPD: −0.136 (−0.228, −0.043) mm, HC: −0.462 (−0.782, −0.142) mm  **Third trimester** [1 μg/m^3^]: BPD: 0.072 (−0.128, 0.273) mm, HC: 0.068 (−0.675, 0.811) mm  **Repeated measures** [1 μg/m^3^]:  BPD: −0.464 (−0.627, −0.301) mm, HC: −1.693 (-2.287, -1.100) mm |  |

**Table S3.** (continued)

|  |  |  |  |  |  |  | **Effect estimates (95% CI) [increment]** | |
| --- | --- | --- | --- | --- | --- | --- | --- | --- |
| **Reference / Cohort** | **Sample size** | **Country** | **Study design** | **GA at outcome assessment** | **Exposure assessment** | **Exposure period** | **Single-pollutant model** | **Multi-pollutant model** |
| Cao et al. 2019  (8) / SMILE | 7965 pregnancies | China | Prospective cohort | 14–40; repeated measurements | Inverse-distance square weighting of routine monitoring data linked to residential addresses | Conception to the last week before US | BPD: −5.82 (−6.88, −4.75) mm [10 μg/m^3^] | Adjusted for SO_2_, NO_2_, PM_10_ and O_3_):  BPD: −5.57 (−6.66, −4.47) mm [10 μg/m^3^] |
| *NO_2_* |  |  |  |  |  |  |  |  |
| Gómez-Herrera et al. 2025 (20) / BiSC * | 754 pregnancies | Spain | Prospective birth cohort study | 222 ± 9 days | LUR, dispersion and hybrid models (main model) linked to home and work addresses and commuting routes | Conception to US | Wider anterior horn of lateral ventricles, wider cisterna magna, larger cerebellar vermis | Wider anterior horn of lateral ventricles (model including NO_2_ and BC) |
| Leung et al. 2023 (3) | 9446 pregnancies from 8241 mothers | USA | Retrospective cohort study | US1: 16–23   US2: 24–31   US3: 32–40 | Machine learning model linked to birth address | Conception to week 37 | **Cumulative exposure [10 ppb]**: BPD z-score US1: −0.05 (−0.10, −0.00), BPD z-score US2: −0.08 (−0.16, −0.01), BPD z-score US3: −0.11 (−0.17, −0.05)  HC z-score US1: −0.03 (−0.08, 0.02), HC z-score US2: −0.08 (−0.16, −0.00), HC z-score  US3: −0.05 (−0.11, 0.01) |  |
| Peterson et al. 2022 (4) / MADRES | 281 pregnancies | USA | Prospective pregnancy cohort | 32±2 wks | Inverse-distance square weighting of routine monitoring data linked to residential histories | Conception to US | **Pregnancy average exposure** [5.6 ppb]:  BPD: 0.001 (-0.9, 0.9) mm  HC: 1.2 (−4.0, 1.6) mm |  |
| Lamichhane et al. 2018  (6) / MOCEH | 426-461 pregnancies | South Korea | Prospective birth cohort study | US1: 12–27 wks US2: 28–40 wks | Residential address linked to land use regression model | Each trimester and whole pregnancy | **T1 exposure** [10 µg/m^3^]: BPD US1: −0.24 (−0.39, −0.08) mm, BPD US2 −0.11 (−0.32, 0.09) mm  **T2 exposure** [10 µg/m^3^]: BPD US1: −0.26 (−0.41, −0.11) mm, BPD US2: −0.22 (−0.48, 0.04) mm  **T3 exposure** [10 µg/m^3^]: BPD US2: −0.19 (−0.42, 0.03) | Adjusted for PM10  **T1 exposure** [10 µg/m^3^]: BPD US1: −0.27 (−0.44, −0.10) mm, BPD US2: −0.11 (−0.32, 0.09) mm  **T2 exposure** [10 µg/m^3^]: BPD US1: −0.26 (−0.42, −0.09) mm, BPD US2: −0.14 (−0.33, 0.05)  **T3 exposure** [10 µg/m^3^]: BPD US2: 0.05 (−0.51, 0.62) mm |
| Wang et al. 2017 (2) / TMCHC | 1001 pregnancies | China | Prospective cohort study | US1: 13±1 wks  US2: 22±1 wks  US3: 32±2 wks | Residential address linked to land use regression model | M1: weeks 0-22   M2: weeks 0-32  and whole pregnancy | **M1 exposure** [10 µg/m^3^]: BPD US2: −0.40 (−0.56, −0.24) mm, HC US2: 1.07 (−1.60, −0.54) mm  **M2 exposure** [10 µg/m^3^]: BPD US3: -0.26 (−0.50, −0.02) mm, HC US2: −0.71 (−1.37, −0.06) mm  **Repeated measures M1 [**10 µg/m^3^]: BPD 0.44 (−0.63 to −0.25) mm, HC −0.95 (−1.45 to −0.45) mm, **M2 [**10 µg/m^3^]: BPD −0.44 (−0.63 to −0.25) mm, HC 1.18 (−1.77 to −0.60) mm |  |

**Table S3.** (continued)

|  |  |  |  |  |  |  | **Effect estimates (95% CI) [increment]** | |
| --- | --- | --- | --- | --- | --- | --- | --- | --- |
| **Reference / Cohort** | **Sample size** | **Country** | **Study design** | **GA at outcome assessment** | **Exposure assessment** | **Exposure period** | **Single-pollutant model** | **Multi-pollutant model** |
| *CO* |  |  |  |  |  |  |  |  |
| Li et al. 2022 (5) / MIH-Hefei | 7419 pregnancies | China | Prospective birth cohort | US1: 14–27 wks  US2: 28–40 wks | Monitoring station located within 3 km of the home address | Trimesters 1, 2 and 3 | **T1 exposure** [0.2 μg/m^3^]: BPD z-score US1: −0.054 (−0.110, 0.002), BPD z-score US2: −0.081 (−0.122, −0.039)  **T2 exposure** [0.2 μg/m^3^]: BPD z-score US2: −0.034 (−0.073, 0.005) |  |
| *BC* |  |  |  |  |  |  |  |  |
| Gómez-Herrera et al. 2025 (20) / BiSC * | 754 pregnancies | Spain | Prospective birth cohort study | 222 ± 9 days | LUR, dispersion and hybrid models (main model) linked to home and work addresses and commuting routes | Conception to US | Wider anterior horn of lateral ventricles, wider cisterna magna, larger cerebellar vermis, shallower sylvian fissure | Wider anterior horn of lateral ventricles, wider cisterna magna,  larger cerebellar vermis, shallower sylvian fissure (ridge regression) |
| *O_3_* |  |  |  |  |  |  |  |  |
| Peterson et al. 2022 (4) / MADRES | 281 pregnancies | USA | Prospective pregnancy cohort | 32±2 wks | Inverse-distance square weighting of routine monitoring data linked to residential histories | Conception to US | **Pregnancy average exposure** [42.3 ppb] BPD: 0.2 (−0.5, 1.0) mm,  HC: −0.7 (−3.0, 1.6) mm |  |
| *SO_2_* |  |  |  |  |  |  |  |  |
| Li et al. 2022 (5) / MIH-Hefei | 7419 pregnancies | China | Prospective birth cohort | US1: 14–27 wks  US2: 28–40 wks | Monitoring station located within 3 km of the home address | Trimesters 1, 2 and 3 | **T1 exposure** [7.8 μg/m^3^]:  BPD z-score US1: −0.022 (−0.089, 0.045), BPD z-score US2: 0.066 (−0.113, −0.019)  **T2 exposure** [7.4 μg/m^3^]:  BPD z-score US2: −0.054 (−0.102, −0.006) |  |

Abbreviations. BPD: biparietal diameter, BC: black carbon, CO: carbon monoxide, GA: gestational age at ultrasound as range or mean ± standard deviation, HC: head circumference, NO_2_: nitrogen dioxide, NO_x_: Nitrogen oxides, O_3_: ozone, PM_10_: particulate matter with a diameter ≤ 10 μm, PM_2.5_: particulate matter with a diameter ≤ 2.5 μm, SO_2_: sulfur dioxide, US: ultrasound.

*This study used transvaginal neurosonography, details on effect estimates are not included in this table due lack of comparability with other studies.

**Table S4.** The relationship between *in utero* air pollution and postnatal MRI brain development during infancy, childhood and adolescence. An overview of study characteristics and main outcomes by pollutant (PM_10_, PM_coarse_, PM_2.5_, PM characteristics, NO_2_/NO_x_) is provided. Structural neuroimaging studies in children and adolescents aged 0-17 years and published between January 1, 2000, and February 25, 2026, were included. Only statistically significant findings are reported (*p* < 0.05). Studies are ordered by year of publication, newest studies first.

| **Publication** | **Sample** |  |  | **Age (years)** | **Exposure time** |  | **MRI modality & measurements included in study** | **Change in outcome with increasing pollution levels** | | |
| --- | --- | --- | --- | --- | --- | --- | --- | --- | --- | --- |
|  |  | **Country** | **Study type** |  |  | **Exposure assessment** |  | **Single-pollutant approach** | **Multi-pollutant approach** |  |
| *PM_10_* |  |  |  |  |  |  |  |  |  |  |
| Kusters et al. 2025 (14) / Generation R | 4243 | The Netherlands | birth cohort | M1: 8.0 (range 6.1–10.7)  M2: 9.9 (range 8.6–12.9)  M3: 13.8 (range 12.6–17.1) | entire pregnancy | LUR model linked to residential addresses | sMRI: WM, cortical GM, CC, CB, thalamus, putamen, pallidum, caudate nucleus, amygdala, hippocampus, nucleus accumbens | **Overall:** Larger CB  **Interaction model:** Larger CB and faster growth, faster hippocampus growth | No significant associations (LASSO) |  |
| Lewandowska et al. 2025 (17) / NeuroSmog | 425 | Poland | ADHD case-control study | 11.32 ± 0.78 | 2nd and 3rd trimester | LUR model linked to residential addresses | dMRI: FA, MD, FBA | No significant associations | - |  |
| Szwed et al. 2025 (22) / ABCD & NeuroSmog | 4713 &  602 | U.S. & Poland | retrospective / child cohort & ADHD case-control study | 10–13 | entire pregnancy | LUR model linked to residential addresses | sMRI: cortical T1w/T2w ratios (proxy for myelin content) | Lower myelin content in the left precuneus in NeuroSmog sample | - |  |
| Kusters et al. 2024 (16) / Generation R | 4108 | The Netherlands | birth cohort | M1: 9–13  M2: 13–17 | entire pregnancy | LUR model linked to residential addresses | dMRI: FA and MD | Lower global FA across M1 and M2, no associations for global MD across M1 and M2 | No significant associations for global FA across M1 and M2 (LASSO) |  |
| Bos et al. 2023 (12) / dHCP | 469 | UK | birth cohort | <1 | entire pregnancy and per trimester | Dispersion model linked to postcodes | sMRI: WM, cortical GM, CB, brainstem, ventricle, deep gray nuclei, extracerebral CSF, amygdala, hippocampus | No significant associations | Larger ventricle, CB, brainstem, extracerebral CSF volume, and smaller cortical GM, amygdala and hippocampus volume (CCA with PM_10_, PM_2.5_ and NO_2_) |  |
| Lubczyńska et al.  2021 (10) / Generation R | 3133 | The Netherlands | birth cohort | 9–12 | entire pregnancy | LUR model linked to residential addresses | sMRI: total brain, cortical GM, subcortical GM, cerebral WM, CC, CB, thalamus, caudate nucleus, putamen, pallidum, hippocampus, amygdala, nucleus accumbens | Larger CB | No significant associations (DSA) |  |

**Table S4.** (continued)

| **Publication** | **Sample** |  |  | **Age (years)** | **Exposure time** |  | **MRI modality & measurements included in study** | **Change in outcome with increasing pollution levels** | | |
| --- | --- | --- | --- | --- | --- | --- | --- | --- | --- | --- |
|  |  | **Country** | **Study type** |  |  | **Exposure assessment** |  | **Single-pollutant approach** | **Multi-pollutant approach** |  |
| Lubczyńska et al. 2020 (18) / Generation R | 2954 | The Netherlands | birth cohort | 9–12 | entire pregnancy | LUR model linked to residential addresses | dMRI: FA and MD | Lower global FA, higher global MD | No significant associations (DSA) |  |
| *PM_coarse_* |  |  |  |  |  |  |  |  |  |  |
| Kusters et al. 2024 (16)/ Generation R | 4108 | The Netherlands | birth cohort | M1: 9–13  M2: 13–17 | entire pregnancy | LUR model linked to residential addresses | dMRI: FA and MD | No associations for global FA and global MD across M1 and M2 | No association for global FA (LASSO) |  |
| Lubczyńska et al. 2021 (10) / Generation R | 3133 | The Netherlands | birth cohort | 9–12 | entire pregnancy | LUR model linked to residential addresses | sMRI: total brain, cortical GM, subcortical GM, cerebral WM, CC, CB, thalamus, caudate nucleus, putamen, pallidum, hippocampus, amygdala, nucleus accumbens | Larger CB, putamen and pallidum | Larger CB (DSA) |  |
| Lubczyńska et al. 2020 (18) / Generation R | 2954 | The Netherlands | birth cohort | 9–12 | entire pregnancy | LUR model linked to residential addresses | dMRI: FA and MD | No significant associations | No significant associations (DSA) |  |
| Guxens et al. 2018 (9) / Generation R | 783 | The Netherlands | birth cohort | 6–10 | entire pregnancy | LUR model linked to residential addresses | sMRI: total brain, cortical GM, cortical WM, subcortical GM, ventricles, cortical thickness | Thinner right lateral orbitofrontal cortex | - |  |
| *PM_2.5_* |  |  |  |  |  |  |  |  |  |  |
| Buthmann et al. 2025 (23) / GUSTO | 325 | Singapore | pregnancy cohort | M1:4.5  M2: 6.0  M3: 7.5  M4: 10.5 | weekly and cumulative exposure | Spatiotemporal interpolation (inverse distance squared weighting of stations within 20 km) | sMRI: hippocampus, amygdala | Late gestation (weeks 36–40) exposure was associated with slower hippocampal volume growth bilaterally between M1 and M4;  week 1-25 faster growth left hippocampal volume;  week 1-13 faster growth right hippocampal volume | - |  |

**Table S4.** (continued)

| **Publication** | **Sample** |  |  | **Age (years)** | **Exposure time** |  | **MRI modality & measurements included in study** | **Change in outcome with increasing pollution levels** | | |
| --- | --- | --- | --- | --- | --- | --- | --- | --- | --- | --- |
|  |  | **Country** | **Study type** |  |  | **Exposure assessment** |  | **Single-pollutant approach** | **Multi-pollutant approach** |  |
| Kusters et al. 2025 (14) / Generation R | 4243 | The Netherlands | birth cohort | M1: 8.0 (range 6.1–10.7)  M2: 9.9 (range 8.6–12.9)  M3: 13.8 (range 12.6–17.1) | entire pregnancy | LUR model linked to residential addresses | sMRI: WM, cortical GM, CC, CB, thalamus, putamen, pallidum, caudate nucleus, amygdala, hippocampus, nucleus accumbens | Repeated measures: larger CB  Interaction with age:  faster growth of hippocampal and CB volume  between M1 and M3 | Faster growth of hippocampal volume between M1 and M3  (LASSO) |  |
| Pujol et al. 2025 (11) / BiSC | 132 | Spain | pregnancy cohort | 29.0 ± 4.3 (days) | M1: 1st trimester  M2: 3dr trimester | LUR with time-activity data for home, commuting and work | sMRI: global myelinated WM, cortical T1w/T2w ratios (proxy for myelin content) | M1 exposure: lower cortical myelination  M2 exposure: lower global myelination | - |  |
| Szwed et al. 2025 (27) (22)/ ABCD | 4713 | U.S. | retrospective / child cohort | 9–10 | entire pregnancy | hybrid spatiotemporal models | sMRI: cortical T1w/T2w ratio (proxy for myelin content) | No significant associations | - |  |
| Kusters et al. 2024 (16) / Generation R | 4108 | The Netherlands | birth cohort | M1: 9–13  M2: 13–17 | entire pregnancy | LUR model linked to residential addresses | dMRI: FA and MD | Lower global FA across M1 and M2, no association for global MD across M1 and M2 | Lower global FA across M1 and M2 (LASSO) |  |
| Bos et al. 2023 (12) / dHCP | 469 | UK | birth cohort | <1 | entire pregnancy and per trimester | Dispersion model linked to postcodes | sMRI: WM, cortical GM, CB, brainstem, ventricle, deep gray nuclei, extracerebral CSF, amygdala, hippocampus | No significant associations | No significant associations (CCA with PM_10_, PM_2.5_ and NO_2_) |  |
| Peterson et al. 2022 (15)/ CCCEH | 332 | USA | pregnancy cohort | 6–14 | entire pregnancy | spatiotemporal air pollution exposure models linked to residential address | sMRI and dMRI: WM, cortical thickness, FA | Thicker lateral temporal, posterior inferior, mesial wall surfaces, thinner dorsal parietal surfaces; smaller inferior parietal lobes, cingulate gyrus WM, larger dorsal convexity, superior frontal gyrus, posterior inferior surface WM; higher FA in basal ganglia, thalamus, and anterior cingulate gyrus | No significant associations (two-pollutant model with PAH and PM_2.5_) |  |

**Table S4.** (continued)

| **Publication** | **Sample** |  |  | **Age (years)** | **Exposure time** |  | **MRI modality & measurements included in study** | **Change in outcome with increasing pollution levels** | | |
| --- | --- | --- | --- | --- | --- | --- | --- | --- | --- | --- |
|  |  | **Country** | **Study type** |  |  | **Exposure assessment** |  | **Single-pollutant approach** | **Multi-pollutant approach** |  |
| Lubczyńska et al.  2021 (10) / Generation R | 3133 | The Netherlands | birth cohort | 9–12 | entire pregnancy | LUR model linked to residential addresses | sMRI: total brain, cortical GM, subcortical GM cerebral WM, CC, CB, thalamus, caudate nucleus, putamen, pallidum, hippocampus, amygdala, nucleus accumbens | Larger CB | No significant associations (DSA) |  |
| Lubczyńska et al. 2020 (18) / Generation R | 2954 | The Netherlands | birth cohort | 9–12 | entire pregnancy | LUR model linked to residential addresses | dMRI: FA and MD | Lower global FA, higher global MD | Lower global FA (DSA) |  |
| Mortamais et al. 2019 (13) / BREATHE | 186 | Spain | retrospective / child cohort | 8–12 | entire pregnancy and per trimester | LUR model linked to residential addresses | sMRI: GM, WM, CC (total, anterior, body and posterior), lateral ventricles | No significant associations |  |  |
| Guxens et al. 2018 (9) / Generation R | 783 | The Netherlands | birth cohort | 6–10 | entire pregnancy | LUR model linked to residential addresses | sMRI: total brain, cortical GM, cortical WM, subcortical GM, ventricles, cortical thickness | Thinner cortex in the right hemisphere of the precuneus, pars opercularis, pars orbitalis, rostral middle frontal and superior frontal region, and in the left hemisphere of the cuneus region |  |  |

**Table S4.** (continued)

| **Publication** | | **Sample** | |  | |  | | **Age (years)** | | **Exposure time** | |  | **MRI modality & measurements included in study** | **Change in outcome with increasing pollution levels** | | |
| --- | --- | --- | --- | --- | --- | --- | --- | --- | --- | --- | --- | --- | --- | --- | --- | --- |
|  |  |  |  | **Country** | | **Study type** | |  |  |  |  | **Exposure assessment** |  | **Single-pollutant approach** | **Multi-pollutant approach** |  |
| *PM characteristics* | | | | | | | | | | | | | | | | |
| Kusters et al. 2025 (14) / Generation R | 4243 | | The Netherlands | | birth cohort | | M1: 8.0 (range 6.1–10.7)  M2: 9.9 (range 8.6–12.9)  M3: 13.8 (range 12.6–17.1) | | entire pregnancy | | LUR model linked to residential addresses | | sMRI: WM, cortical GM, CC, CB, thalamus, putamen, pallidum, caudate nucleus, amygdala, hippocampus, nucleus accumbens | **Repeated measures:**  PM_2.5_ absorbance: larger CB, smaller CC, Zn: larger CB, smaller CC, PAH: larger CB, OC: smaller CC, larger hippocampus, Si: larger amygdala  **Interaction with age:**  PM_2.5_ absorbance: faster CB and hippocampal growth, Cu: smaller hippocampal volume at M1 and faster growth of hippocampal volume and CB between M1 and M3, Fe: faster hippocampal growth, slower caudate nucleus growth, Si: faster hippocampal growth, smaller amygdala at M1, Zn: larger CB at M1, PAH: faster hippocampal growth  OC: larger hippocampal volume at M1, OPDTT: faster thalamus growth, OPESR: faster hippocampal growth, slower caudate nucleus growth | Cu: faster growth of hippocampal volume between M1 and M3 (LASSO) |  |
| Pujol et al. 2025 (11) / BiSC | 132 | | Spain | | pregnancy cohort | | 29.0 ± 4.3 (days) | | M1: 1st trimester  M2: 3dr trimester | | Land Use Regression with time-activity data for home, commuting and work | | sMRI: global myelinated WM, cortical T1w/T2w ratios (proxy for myelin content) | Fe and Cu: lower cortical myelination for M2 exposure | No significant associations (models adjusted for PM_2.5_) |  |
| Yang et al. 2025 (24) | 165 | | U.S. | | birth cohort | | 17 ± 1.24 (range 13-17) | | Third trimester | | PAH-DNA adducts in maternal blood | | sMRI: hippocampus and hippocampal subfields | Smaller left hippocampal CA2/3 vol (detectable PAH-DNA adducts vs. not detectable) | - |  |
| Kusters et al. 2024 (16) / Generation R |  | | The Netherlands | | birth cohort | | M1: 9–13  M2: 13–17 | | entire pregnancy | | LUR model linked to residential addresses l | | dMRI: FA and MD | PM_2.5_ absorbance: lower global FA across M1 and M2 | PM_2.5_ absorbance: no significant associations for global FA across M1 and M2 (LASSO) |  |

**Table S4.** (continued)

| **Publication** | | **Sample** | |  | |  | | **Age (years)** | | **Exposure time** | |  | **MRI modality & measurements included in study** | **Change in outcome with increasing pollution levels** | | |
| --- | --- | --- | --- | --- | --- | --- | --- | --- | --- | --- | --- | --- | --- | --- | --- | --- |
|  |  |  |  | **Country** | | **Study type** | |  |  |  |  | **Exposure assessment** |  | **Single-pollutant approach** | **Multi-pollutant approach** |  |
| Peterson et al. 2022 (15) / CCCEH | 332 | | USA | | pregnancy cohort | | 6–14 | | a 48-hour period in the third trimester of pregnancy | | personal air monitoring | | sMRI and dMRI: WM, cortical thickness, FA | PAH: thicker lateral temporal, posterior inferior, mesial wall surfaces, thinner dorsal parietal surfaces; smaller inferior parietal lobes, cingulate gyrus, and larger dorsal convexity, superior frontal gyrus, and posterior inferior brain surface WM; higher FA in basal ganglia, thalamus, and anterior cingulate gyrus |  |  |
| Margolis et al. 2022 (25) / Sibling-Hermanos birth cohort | 37 | | USA | | child recruitment from birth cohort | | 7–9 | | a 48-hour period in the third trimester of pregnancy | | personal air monitoring | | sMRI: hippocampus, and hippocampal subfields | PAH: no significant associations |  |  |
| Lubczyńska et al. 2021 (10) / Generation R | 3133 | | The Netherlands | | birth cohort | | 9–12 | | entire pregnancy | | LUR model linked to residential addresses | | sMRI: total brain, cortical GM, subcortical GM, cerebral WM, CC, CB, thalamus, caudate nucleus, putamen, pallidum, hippocampus, amygdala, nucleus accumbens, cortical thickness | PM_2.5_ absorbance: larger CB, OP_ESR_: smaller CC, PAH, B[a]P, and Cu: smaller hippocampus, Si: larger amygdala, OC: thinner cortex of the right postcentral gyrus, PM_2.5_ absorbance and CU: thinner cortex right in rostral middle frontal gyrus | OP_ESR_: smaller CC  OP_DTT_: smaller CB  PAHs: smaller hippocampus  OC, PAHs, Si: smaller amygdala (DSA) |  |
| Lubczyńska et al. 2020 (18) / Generation R | 2954 | | The Netherlands | | birth cohort | | 9–12 | | entire pregnancy | | LUR model linked to residential addresses | | dMRI: FA and MD | PM_2.5_ absorbance: lower global FA  PM_2.5_ absorbance: Cu, Fe, Si, OP_ESR_, UFP: higher global MD | PAH: higher global FA  Si: higher global MD (DSA) |  |
| Guxens et al. 2018 (9) / Generation R | 783 | | The Netherlands | | birth cohort | | 6–10 | | entire pregnancy | | LUR model linked to residential addresses | | sMRI: total brain, cortical GM, cortical WM, subcortical GM, ventricles, cortical thickness | PM_2.5_ absorbance: thinner left fusiform cortex |  |  |
| Peterson et al. 2015 (26) / CCCEH | 40 | | USA | | pregnancy cohort | | 7–9 | | a 48-hour period in the third trimester of pregnancy | |  | | sMRI: cerebral and WM surface | PAH: smaller left lateral, mesial, dorsal, and ventral WM surfaces |  |  |

**Table S4.** (continued)

| **Publication** | | **Sample** | |  | |  | | **Age (years)** | | **Exposure time** | |  | **MRI modality & measurements included in study** | **Change in outcome with increasing pollution levels** | | |
| --- | --- | --- | --- | --- | --- | --- | --- | --- | --- | --- | --- | --- | --- | --- | --- | --- |
|  |  |  |  | **Country** | | **Study type** | |  |  |  |  | **Exposure assessment** |  | **Single-pollutant approach** | **Multi-pollutant approach** |  |
| *NO_2_ / NO_x_* |  | |  | |  | |  | |  | |  | |  |  |  |  |
| Kusters et al. 2025 (14) / Generation R | 4243 | | The Netherlands | | birth cohort | | M1: 8.0 (range 6.1–10.7)  M2: 9.9 (range 8.6–12.9)  M3: 13.8 (range 12.6–17.1) | | entire pregnancy | | LUR model linked to residential addresses | | sMRI: WM, cortical GM, CC, CB, thalamus, putamen, pallidum, caudate nucleus, amygdala, hippocampus, nucleus accumbens | Repeated measures:  Smaller CB (NO_x_), smaller CC (NO_2_, NO_x_)  Interaction with age:  faster CB growth (NO_2_), smaller CC at M1 (NO_x_) | No significant associations (LASSO) |  |
| Lewandowska et al. 2025 (17) / NeuroSmog | 425 | | Poland | | ADHD case-control study | | 11.32 ± 0.78 | | 2nd and 3rd trimester | | LUR model linked to residential addresses | | dMRI: FA, MD, FBA | No significant associations (NO_2_) | - |  |
| Szwed et al. 2025 (22)/ ABCD and NeuroSmog | 4713 &  602 | | U.S. & Poland | | retrospective / child cohort & ADHD case-control study | | 10–13 | | entire pregnancy | | LUR model linked to residential addresses | | sMRI: cortical T1w/T2w ratios (proxy for myelin content) | No significant associations | - |  |
| Kusters et al. 2024 (16) / Generation R | 4108 | | The Netherlands | | birth cohort | | M1: 9–13  M2: 13–17 | | entire pregnancy | | LUR model linked to residential addresses | | dMRI: FA and MD | No significant associations (NO_2_ and NO_x_) for global FA and global MD across M1 and M2 | No significant association for global FA (LASSO) |  |
| Bos et al. 2023 (12) / dHCP | 469 | | UK | | birth cohort | | <1 | | entire pregnancy and per trimester | | Dispersion model linked to postcodes | | sMRI: WM, cortical GM, CB, brainstem, ventricle, deep gray nuclei, extracerebral CSF, amygdala, hippocampus | No significant associations (NO_2_) | Smaller ventricle volume, CB, brainstem, extracerebral CSF volume, and larger cortical GM, amygdala & hippocampus (CCA with PM_10_ and PM_2.5_ and NO_2_) |  |
| Lubczyńska et al. 2021 (10) / Generation R | 3133 | | The Netherlands | | birth cohort | | 9–12 | | entire pregnancy | | LUR model linked to residential addresses | | sMRI: total brain, cortical GM, subcortical GM, cerebral WM, CC, CB, thalamus, caudate nucleus, putamen, pallidum, hippocampus, amygdala, nucleus accumbens | No significant associations (NO_2_ and NO_x_) | No significant associations (DSA) |  |
| Lubczyńska et al. 2020 (18) / Generation R | 2954 | | The Netherlands | | birth cohort | | 9–12 | | entire pregnancy | | LUR model linked to residential addresses | | dMRI: FA and MD | Higher global MD (NO_2_ and NO_x_); lower global FA (NO_x_) | No significant associations (NO_x_ and NO_2_) (DSA) |  |

**Table S4.** (continued)

| **Publication** | | **Sample** | |  | |  | | **Age (years)** | | **Exposure time** | |  | **MRI modality & measurements included in study** | **Change in outcome with increasing pollution levels** | | |
| --- | --- | --- | --- | --- | --- | --- | --- | --- | --- | --- | --- | --- | --- | --- | --- | --- |
|  |  |  |  | **Country** | | **Study type** | |  |  |  |  | **Exposure assessment** |  | **Single-pollutant approach** | **Multi-pollutant approach** |  |
| Guxens et al. 2018 (9) / Generation R | 783 | | The Netherlands | | birth cohort | | 6–10 | | entire pregnancy | | LUR model linked to residential addresses | | sMRI: total brain, cortical GM, cortical WM, subcortical GM, ventricles, cortical thickness | No significant associations (NO_2_) |  |  |

Abbreviations: B[a]P: Benzo(a)pyrene, CB: cerebellum, CC: corpus callosum, CCA: canonical correlation analysis, CSF: cerebrospinal fluid, Cu: copper, dMRI: diffusion MRI, DSA: Deletion/Substitution/Addition, FA: fractional anisotropy, FBA: fixel-based analysis, Fe: iron, , OC: organic carbon, GM: gray matter, WM: white matter, MD: mean diffusivity, MRI: magnetic resonance imaging, NO_2_: nitrogen dioxide, NO_x_: nitrogen oxides, OP_ESR_: oxidative potential measured by electron spin, OP_DTT_: oxidative potential measured by dithiothreitol, PAH: polycyclic aromatic hydrocarbons, PM: particulate matter, PM_10_: PM with a diameter ≤ 10 μm, PM_coarse_: PM with a diameter of 2.5 - 10 μm, PM_2.5_: PM with a diameter ≤ 2.5 μm, Si: silicon, sMRI: structural MRI, UFP: ultra fine particles.

**Table S5.**  Cohort-specific quality assessment by the Newcastle-Ottawa scale for the ultrasound studies investigating the relationship between *in utero* air pollution exposure and fetal brain development as published between January 2017 and February 2026. Studies are ordered by year of publication, newest studies first.

|  | Gómez-Herrera et al. 2025 (20) | Chen et al. 2023 (7) | Leung et al.  2023 ​(3) | Peterson et al.  2022 ​(4) | Li et al. 2022 ​(5) | Cao et al.  2021 ​(21)​ | Zhao et al.  2021 ​(1) | Cao et al. 2019 ​(8) | Lamichhane et al. 2018 ​(6) | Zhao et al.  2018 (19) | Wang et al.  2017 ​(2) |
| --- | --- | --- | --- | --- | --- | --- | --- | --- | --- | --- | --- |
| **Selection** |  |  |  |  |  |  |  |  |  |  |  |
| 1) Representativeness of the exposed cohort (1 point) |  |  |  |  |  |  |  |  |  |  |  |
| a) truly representative of the average pregnant woman in the community * |  |  |  |  |  |  |  |  |  |  |  |
| b) somewhat representative of the average pregnant woman in the community * | X | X | X |  | X | X |  | X | X | X | X |
| c) selected group |  |  |  | X |  |  |  |  |  |  |  |
| d) no description of the derivation of the cohort |  |  |  |  |  |  | X |  |  |  |  |
| 2) Selection of the non-exposed cohort (1 point) |  |  |  |  |  |  |  |  |  |  |  |
| a) drawn from the same community as the exposed cohort * | X | X | X | X | X | X | X | X | X | X | X |
| b) drawn from a different source |  |  |  |  |  |  |  |  |  |  |  |
| c) no description of the derivation of the non-exposed cohort |  |  |  |  |  |  |  |  |  |  |  |
| 3) Ascertainment of exposure (1 point) |  |  |  |  |  |  |  |  |  |  |  |
| a) high-resolution air pollution model linked to residential address * | X | X | X |  |  |  | X |  |  |  | X |
| b) (interpolated) central air quality monitoring site data * |  |  |  | X | X | X |  | X | X | X |  |
| c) no description |  |  |  |  |  |  |  |  |  |  |  |
| 4) Demonstration that outcome of interest was not present at start of study (1 point) |  |  |  |  |  |  |  |  |  |  |  |
| a) yes * |  |  |  |  |  |  |  |  |  |  |  |
| b) no | X | X | X | X | X | X | X | X | X | X | X |
| **Comparability** |  |  |  |  |  |  |  |  |  |  |  |
| 1) Comparability of cohorts on the basis of the design or analysis (1 point) |  |  |  |  |  |  |  |  |  |  |  |
| a) study controls for maternal age * |  | X | X | X | X | X | X | X | X | X | X |
| b) study controls for maternal smoking * | X | X | X |  |  |  | X |  | X | X | X |
| c) study controls for socio-economic status * | X | X | X | X | X | (X) | X | (X) | X | X | X |

**Table S5.** (continued)

|  | Gómez-Herrera et al. 2025 (20) | Chen et al. 2023 (7) | Leung et al.  2023 ​(3) | Peterson et al.  2022 ​(4) | Li et al. 2022 ​(5) | Cao et al.  2021 ​(21)​ | Zhao et al.  2021 ​(1) | Cao et al. 2019 ​(8) | Lamichhane et al. 2018 ​(6) | Zhao et al.  2018 (19) | Wang et al.  2017 ​(2) |
| --- | --- | --- | --- | --- | --- | --- | --- | --- | --- | --- | --- |
| **Outcome** |  |  |  |  |  |  |  |  |  |  |  |
| 1) Assessment of outcome (1 point) |  |  |  |  |  |  |  |  |  |  |  |
| a) independent blind assessment * | X | X | X | X | X | X | X | X | X | X | X |
| c) self-report |  |  |  |  |  |  |  |  |  |  |  |
| d) no description |  |  |  |  |  |  |  |  |  |  |  |
| 2) Follow-up long enough for outcomes to occur (1 point) |  |  |  |  |  |  |  |  |  |  |  |
| a) yes (select an adequate follow up period for outcome of interest) * | X | X | X | X | X | X | X | X | X | X | X |
| b) no |  |  |  |  |  |  |  |  |  |  |  |
| 3) Adequacy of follow up of cohorts (1 point) |  |  |  |  |  |  |  |  |  |  |  |
| a) complete follow up - all subjects accounted for * |  |  | X |  |  |  |  |  |  |  |  |
| b) subjects lost to follow up unlikely to introduce bias - small number lost - < 5% or no differential missingness * | X | X |  |  |  |  |  |  |  |  |  |
| c) subject lost to follow up may introduce bias - large number lost > 10% - differential missingness |  |  |  |  | X | X |  |  |  |  |  |
| d) no statement |  |  |  | X |  |  | X | X | X | X | X |
| **Total Score (Stars out of a max. 8)** | **8** | **8** | **8** | **6** | **7** | **7** | **6** | **7** | **7** | **7** | **7** |

**Table S6.**  Cohort-specific quality assessment by the Newcastle-Ottawa scale for the 15 studies investigating the relationship between *in utero* air pollution exposure and postnatal brain development as published between January 2017 and February 2026. Studies are ordered by year of publication, newest studies first.

|  | Buthmann et al. 2025 (23) | Kusters et al. 2025 (14) | Lewandowska et al. 2025 (17) | Pujol et al. 2025 (11) | Szwed et al. 2025 (22) | Yang et al. 2025 (24) | Kusters et al. 2024 (16) | Bos et al. 2023 (12) | Margolis et al. 2022 (25) | Peterson et al. 2022 (15) | Lubczyńska et al. 2021 (10) | Lubczyńska et al. 2020 (18) | Mortamais et al. 2019 (13) | Guxens et al. 2018 (9) | Peterson et al. 2015 (26) |
| --- | --- | --- | --- | --- | --- | --- | --- | --- | --- | --- | --- | --- | --- | --- | --- |
| **Selection** |  |  |  |  |  |  |  |  |  |  |  |  |  |  |  |
| 1) Representativeness of the exposed cohort (1 point) |  |  |  |  |  |  |  |  |  |  |  |  |  |  |  |
| a) truly representative of the average mother/ child in the community * |  |  |  |  |  |  |  |  |  |  |  |  |  |  |  |
| b) somewhat representative of the average mother/ child in the community * | X | X |  | X | X |  | X | X |  |  | X | X | X | X |  |
| c) selected group |  |  | X |  |  | X |  |  | X | X |  |  |  |  | X |
| d) no description of the derivation of the cohort |  |  |  |  |  |  |  |  |  |  |  |  |  |  |  |
| 2) Selection of the non-exposed cohort (1 point) |  |  |  |  |  |  |  |  |  |  |  |  |  |  |  |
| a) drawn from the same community as the exposed cohort * | X | X | X | X | X | X | X | X | X | X | X | X | X | X | X |
| b) drawn from a different source |  |  |  |  |  |  |  |  |  |  |  |  |  |  |  |
| c) no description of the derivation of the non-exposed cohort |  |  |  |  |  |  |  |  |  |  |  |  |  |  |  |
| 3) Ascertainment of exposure (1 point) |  |  |  |  |  |  |  |  |  |  |  |  |  |  |  |
| a) high-resolution air pollution model linked to maternal residential address * |  | X | X | X | X |  | X | X |  |  | X | X | X | X |  |
| b) (interpolated) central air quality monitoring site data * | X |  |  |  | X |  |  |  |  |  |  |  |  |  |  |
| c) personal monitoring * |  |  |  |  |  | X |  |  | X | X |  |  |  |  | X |
| d) no description |  |  |  |  |  |  |  |  |  |  |  |  |  |  |  |
| 4) Exposure period precedes outcome measurement (1 point) |  |  |  |  |  |  |  |  |  |  |  |  |  |  |  |
| a) yes * | X | X | X | X | X | X | X | X | X | X | X | X | X | X | X |
| b) no |  |  |  |  |  |  |  |  |  |  |  |  |  |  |  |

**Table S6.** (continued).

|  | Buthmann et al. 2025 (23) | Kusters et al. 2025 (14) | Lewandowska et al. 2025 (17) | Pujol et al. 2025 (11) | Szwed et al. 2025 (22) | Yang et al. 2025 (24) | Kusters et al. 2024 (16) | Bos et al. 2023 (12) | Margolis et al. 2022 (25) | Peterson et al. 2022 (15) | Lubczyńska et al. 2021 (10) | Lubczyńska et al. 2020 (18) | Mortamais et al. 2019 (13) | Guxens et al. 2018 (9) | Peterson et al. 2015 (26) |
| --- | --- | --- | --- | --- | --- | --- | --- | --- | --- | --- | --- | --- | --- | --- | --- |
| **Comparability** |  |  |  |  |  |  |  |  |  |  |  |  |  |  |  |
| 1) Comparability of cohorts on the basis of the design or analysis (1 point) |  |  |  |  |  |  |  |  |  |  |  |  |  |  |  |
| a) study controls for child age * |  | X | X | X | X | X | X | X | X | X | X | X | X | X | X |
| b) study controls for maternal smoking * |  | X |  |  |  |  | X | X | X | X | X | X | X | X |  |
| c) study controls for socio-economic status * | X | X | X | X | X | X | X | (X) |  | X | X | X | X | X | X |
| **Outcome** |  |  |  |  |  |  |  |  |  |  |  |  |  |  |  |
| 1) Assessment of outcome (1 point) |  |  |  |  |  |  |  |  |  |  |  |  |  |  |  |
| a) independent blind assessment * | X | X | X | X | X | X | X | X | X | X | X | X | X | X | X |
| c) self-report |  |  |  |  |  |  |  |  |  |  |  |  |  |  |  |
| d) no description |  |  |  |  |  |  |  |  |  |  |  |  |  |  |  |
| 2) Was follow-up long enough for outcomes to occur (1 point) |  |  |  |  |  |  |  |  |  |  |  |  |  |  |  |
| a) yes (select an adequate follow up period for outcome of interest) * | X | X | X | X | X | X | X | X | X | X | X | X | X | X | X |
| b) no |  |  |  |  |  |  |  |  |  |  |  |  |  |  |  |
| 3) Adequacy of follow up of cohorts (1 point) |  |  |  |  |  |  |  |  |  |  |  |  |  |  |  |
| a) complete follow up - all subjects accounted for * |  |  |  |  |  |  |  |  |  |  |  |  |  |  |  |
| b) subjects lost to follow up unlikely to introduce bias - small number lost - < 5% or no differential missingness * |  | X |  |  |  |  | X |  |  |  | X | X | X | X |  |
| c) subject lost to follow up may introduce bias - large number lost > 10% - differential missingness | X |  |  |  |  | X |  |  |  | X |  |  |  |  | X |
| d) no statement |  |  | X | X | X |  |  | X | X |  |  |  |  |  |  |
| **Total Score (Stars out of a max. 8)** | **7** | **8** | **6** | **7** | **6** | **6** | **8** | **7** | **6** | **6** | **8** | **8** | **8** | **8** | **6** |

**References**

1. Zhao Y, Wang P, Zhou Y, Xia B, Zhu Q, Ge W, et al. Prenatal fine particulate matter exposure, placental DNA methylation changes, and fetal growth. Environ Int. 2021;147:106313.

2. Wang W, Zhong C, Huang L, Zhou X, Chen R, Wu J, et al. Prenatal NO(2) exposure and ultrasound measures of foetal growth: a prospective cohort study in Wuhan, China. Occup Environ Med. 2017;74(3):204–10.

3. Leung M, Modest AM, Hacker MR, Wylie BJ, Wei Y, Schwartz J, et al. Traffic-Related Air Pollution and Ultrasound Parameters of Fetal Growth in Eastern Massachusetts. Am J Epidemiol. 2023;192(7):1105–15.

4. Peterson AK, Habre R, Niu Z, Amin M, Yang T, Eckel SP, et al. Identifying pre-conception and pre-natal periods in which ambient air pollution exposure affects fetal growth in the predominately Hispanic MADRES cohort. Environ Health. 2022;21(1):115.

5. Li L, Yin W, Wang P, Ma S, Zhou M, Li P, et al. The role of cortisol in the association between prenatal air pollution and fetal growth: A prospective cohort study. Environ Res. 2022;212(Pt B):113250.

6. Lamichhane DK, Ryu J, Leem JH, Ha M, Hong YC, Park H, et al. Air pollution exposure during pregnancy and ultrasound and birth measures of fetal growth: A prospective cohort study in Korea. Sci Total Environ. 2018;619-620:834–41.

7. Chen WJ, Rector AM, Guxens M, Iniguez C, Swartz MD, Symanski E, et al. Susceptible windows of exposure to fine particulate matter and fetal growth trajectories in the Spanish INMA (INfancia y Medio Ambiente) birth cohort. Environ Res. 2023;216(Pt 2):114628.

8. Cao Z, Meng L, Zhao Y, Liu C, Yang Y, Su X, et al. Maternal exposure to ambient fine particulate matter and fetal growth in Shanghai, China. Environ Health. 2019;18(1):49.

9. Guxens M, Lubczynska MJ, Muetzel RL, Dalmau-Bueno A, Jaddoe VWV, Hoek G, et al. Air Pollution Exposure During Fetal Life, Brain Morphology, and Cognitive Function in School-Age Children. Biol Psychiatry. 2018;84(4):295–303.

10. Lubczynska MJ, Muetzel RL, El Marroun H, Hoek G, Kooter IM, Thomson EM, et al. Air pollution exposure during pregnancy and childhood and brain morphology in preadolescents. Environ Res. 2021;198:110446.

11. Pujol J, Martinez-Vilavella G, Gomez-Herrera L, Rivas I, Gomez-Roig MD, Llurba E, et al. Unraveling the impact of prenatal air pollution for neonatal brain maturation. Environ Int. 2025;204:109801.

12. Bos B, Barratt B, Batalle D, Gale-Grant O, Hughes EJ, Beevers S, et al. Prenatal exposure to air pollution is associated with structural changes in the neonatal brain. Environ Int. 2023;174:107921.

13. Mortamais M, Pujol J, Martinez-Vilavella G, Fenoll R, Reynes C, Sabatier R, et al. Effects of prenatal exposure to particulate matter air pollution on corpus callosum and behavioral problems in children. Environ Res. 2019;178:108734.

14. Kusters MSW, Binter AC, Muetzel RL, Lopez-Vicente M, Petricola S, Tiemeier H, et al. Outdoor residential air pollution exposure and the development of brain volumes across childhood: A longitudinal study. Environ Pollut. 2025;373:126078.

15. Peterson BS, Bansal R, Sawardekar S, Nati C, Elgabalawy ER, Hoepner LA, et al. Prenatal exposure to air pollution is associated with altered brain structure, function, and metabolism in childhood. J Child Psychol Psychiatry. 2022;63(11):1316–31.

16. Kusters MSW, Lopez-Vicente M, Muetzel RL, Binter AC, Petricola S, Tiemeier H, et al. Residential ambient air pollution exposure and the development of white matter microstructure throughout adolescence. Environ Res. 2024;262(Pt 2):119828.

17. Lewandowska P, Bajada CJ, Mysak Y, Domagalik A, Kossowski B, Baumbach C, et al. The Impact of Early Life Exposure to Air Pollution on the Brain: A Diffusion MRI Study in 10-13-Year-Old Children With and Without ADHD Diagnosis. Hum Brain Mapp. 2025;46(14):e70306.

18. Lubczynska MJ, Muetzel RL, El Marroun H, Basagana X, Strak M, Denault W, et al. Exposure to Air Pollution during Pregnancy and Childhood, and White Matter Microstructure in Preadolescents. Environ Health Perspect. 2020;128(2):27005.

19. Zhao N, Qiu J, Ma S, Zhang Y, Lin X, Tang Z, et al. Effects of prenatal exposure to ambient air pollutant PM10 on ultrasound-measured fetal growth. Int J Epidemiol. 2018;47(4):1072–81.

20. Gomez-Herrera L, Zhao Y, Rivas I, Eixarch E, Dominguez-Gallardo C, Galmes T, et al. Air pollution and fetal brain morphological development: a prospective cohort study. Lancet Planet Health. 2025;9(6):e480–e90.

21. Cao ZJ, Zhao Y, Wang SM, Zhang DL, Zhou YC, Liu WN, et al. Prenatal exposure to fine particulate matter and fetal growth: a cohort study from a velocity perspective. Chemosphere. 2021;262:128404.

22. Szwed M, de Jesus AV, Kossowski B, Ahmadi H, Rutkowska E, Mysak Y, et al. Air pollution and cortical myelin T1w/T2w ratio estimates in school-age children from the ABCD and NeuroSmog studies. Dev Cogn Neurosci. 2025;73:101538.

23. Buthmann JL, Benmarhnia T, Huang JY, Huang P, Miller JG, Uy JP, et al. Exposure to Fine Particulate Matter During Pregnancy Is Associated With Hippocampal Development in Offspring. Biol Psychiatry Glob Open Sci. 2025;5(4):100490.

24. Yang H, Cohen JW, Pagliaccio D, Ramphal B, Rauh V, Perera F, et al. Prenatal exposure to polycyclic aromatic hydrocarbons, reduced hippocampal subfield volumes, and word reading. Dev Cogn Neurosci. 2025;72:101508.

25. Margolis AE, Cohen JW, Ramphal B, Thomas L, Rauh V, Herbstman J, et al. Prenatal Exposure to Air Pollution and Early-Life Stress Effects on Hippocampal Subregional Volumes and Associations With Visuospatial Reasoning. Biol Psychiatry Glob Open Sci. 2022;2(3):292–300.

26. Peterson BS, Rauh VA, Bansal R, Hao X, Toth Z, Nati G, et al. Effects of prenatal exposure to air pollutants (polycyclic aromatic hydrocarbons) on the development of brain white matter, cognition, and behavior in later childhood. JAMA Psychiatry. 2015;72(6):531–40.
